# Semisynthesis and Evaluation of Anti-Inflammatory Activity of the Cassane-Type Diterpenoid Taepeenin F and of Some Synthetic Intermediates

**DOI:** 10.1021/acs.jnatprod.2c00578

**Published:** 2022-10-10

**Authors:** Houda Zentar, Fatin Jannus, Pilar Gutierrez, Marta Medina-O’Donnell, José Antonio Lupiáñez, Fernando J. Reyes-Zurita, Enrique Alvarez-Manzaneda, Rachid Chahboun

**Affiliations:** † Departamento de Química Orgánica, Facultad de Ciencias, Instituto de Biotecnología, Universidad de Granada, 18071 Granada, Spain; ¥ Departamento de Bioquímica y Biología Molecular I, Facultad de Ciencias, Universidad de Granada, 18071 Granada, Spain

## Abstract

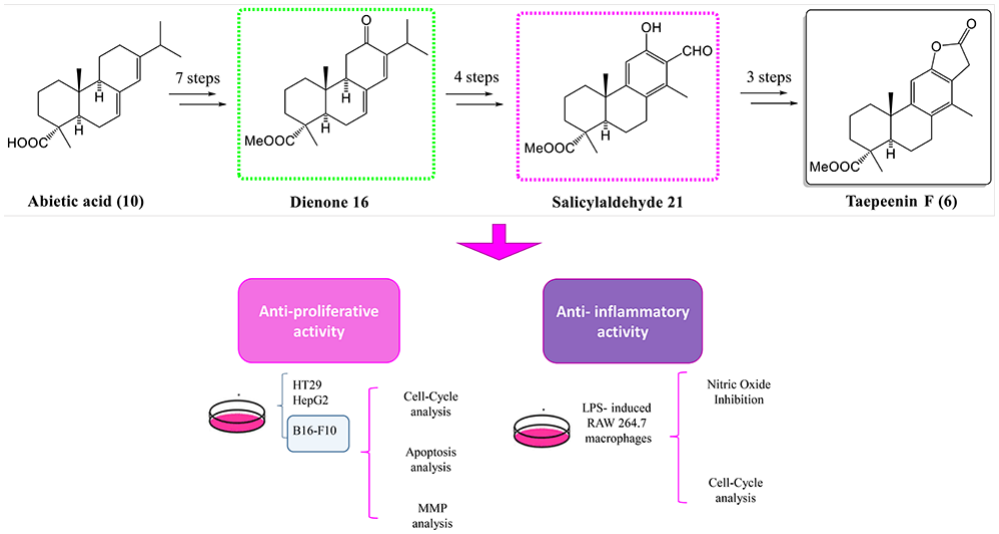

A new strategy for the semisynthesis of the aromatic cassane-type diterpene taepeenin F (**6**) is reported. The introduction of the methyl group at C-14, characteristic of the target compound, was achieved via dienone **13**, easily prepared from abietic acid (**10**), the major compound in renewable rosin. Biological assays of selected compounds are reported. The antiproliferative activity against HT29, B16-F10, and HepG2 tumor cell lines has been investigated. Salicylaldehyde **21** was the most active compound (IC_50_ = 7.72 μM). Products **16** and **21** displayed apoptotic effects in B16-F10 cells, with total apoptosis rates of 46 and 38.4%, respectively. This apoptotic process involves a significant arrest of the B16-F10 cell cycle, blocking the G0/G1 phase. Dienone **16** did not cause any loss of the mitochondrial membrane potential (MMP), while salicylaldehyde **21** caused a partial loss of the MMP. The anti-inflammatory activity of the selected compounds was investigated with the LPS-stimulated RAW 264.7 macrophages. All compounds showed potent NO inhibition, with percentages between 80 and 99% at subcytotoxic concentrations. Dienone **16** inhibited LPS-induced differentiation of RAW 264.7 cells, by increasing the proportion of cells in the S phase. In addition, salicylaldehyde **21** had effects on the cell cycle, recovering the cells from the G0/G1 full arrest produced in response to LPS action.

Cassanes are a family of natural diterpenes isolated from medicinal plants belonging especially the *genus Caesalpinia*.^1^ The interest of this group of metabolites lies not only in their great structural variety but also in the important and promising biological activities they exhibit.^2^ Cassane-type furan diterpenes represent an important subgroup of these metabolites, characterized by a furanic or butenolide ring fused at C-12 and C-13 of the cassane skeleton. Some compounds of this class of diterpenes are sucutiniranes C (**1**) and D (**2**), which have been isolated from *Bowdichia nitida*, an indigenous plant of South America used for the treatment of rheumatic, feverish, and gouty conditions.^3^ (5α)-Vouacapane-8(14),9(11)-diene (**4**), isolated from *Caesalpinia Crista*, with an NO inhibition ratio of 34.5%,^4−7^ and taepeenin F (**6**) with the γ-lactone group fused have been isolated from the stem and roots of *C. crista*.^8^ In addition, caesmimotam A (**7a**) and B (**7b**), two unusual cassane diterpenes featuring a basic furanoditerpenoid skeleton with a lactam ring fused, have been isolated from *C. mimosoides*.^9^ On the other hand, tricyclic cassane diterpenoids have been also reported for their biological activities, for instance, caesalpin A **(8**), which presents significant efects with IC_50_ values of 4.7 and 2.1 μM, for HepG-2 and MCF-7 cancer cell lines, respectively.^10^ The norcassane diterpene 16-norcaesalpinin C (**9**) displays antimalarial activity against the malaria parasite *Plasmodium falciparum* FCR-3/A2 clone in vitro, with an IC_50_ value of 5.0 μM.^11^

However, even with the evident interest of this type of compound, few synthetic studies of aromatic cassane-type terpenes have been developed.^12,13^ Pitsinos et al. prepared a series of 14-desmethyl derivatives and second-generation analogues of taepeenin D (**5**), exhibiting significant Hh/GLI-mediated transcription inhibitory activity and selective cytotoxicity against cancer cells with increased Hedgehog (Hh) signaling levels.^14^ Our research group reported the first enantioselective synthesis of cassane benzofuran, benthaminin 1 (**3**) from *trans*-communic acid.^15^ Our group also described the first synthesis of taepeenin F (**6**) starting from dehydroabietic acid,^16^ but the synthetic procedure was quite long (18 steps). We also reported the first synthesis of (5α)-vouacapane-8(14),9(11)-diene (**4**) from (+)-sclareolide.^17^
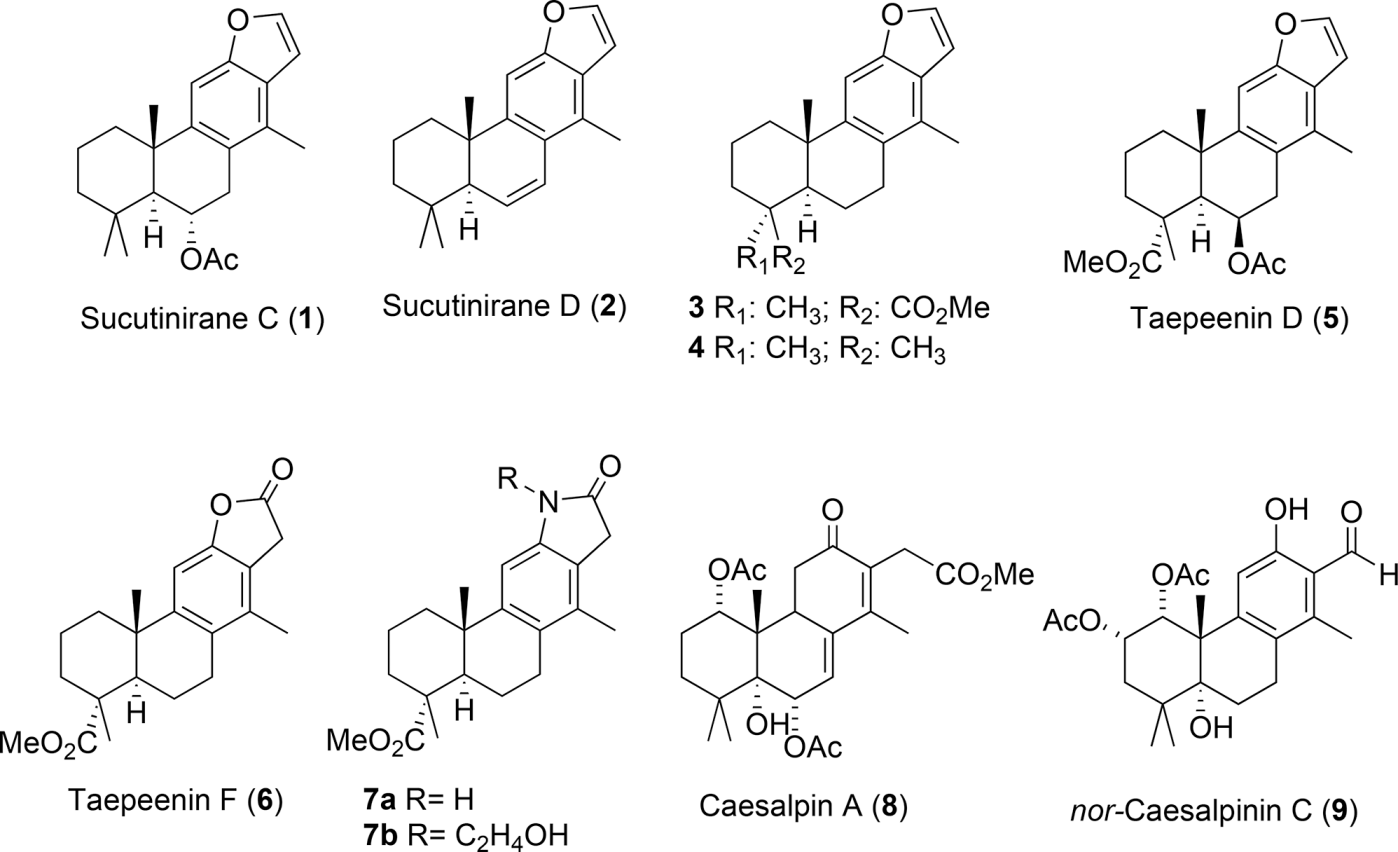


All these data reveal the importance of the use of abundant natural products for the stereoselective synthesis of cassanes. In this regard, synthetic strategies toward taepeenins D (**5**) and F (**6**) were proposed, using dehydroabietic acid as the most suitable starting material; however, the obtained results showed the great difficulty for the introduction of the methyl group at C-14 through this starting material.^14,16,17^ Structurally, abietic acid (**10**), the most abundant of resin acids, seems to be the most suitable starting product to approach the semisynthesis of molecules with a cassane skeleton such as taeepenin D (**5**) or F (**6**). The presence of the Δ^13^ double bond could be the key to the introduction of the methyl group at C-14. Other advantages of using this raw material are its commercial availability and its economic value.

Herein, a new semisynthesis of taepeenin F (**6**) from abietic acid (**10**) is proposed. In short, we have evaluated its antiproliferative activity with other synthesized intermediate compounds, in the tumor cell lines HT29, B16-F10, and HepG-2. Finally, the NO inhibitory activity of these compounds is examined in the murine macrophage cell line RAW 264.7 induced by lipopolysaccharides (LPS).

## Results and Discussion

### Chemistry

**Scheme 1:**
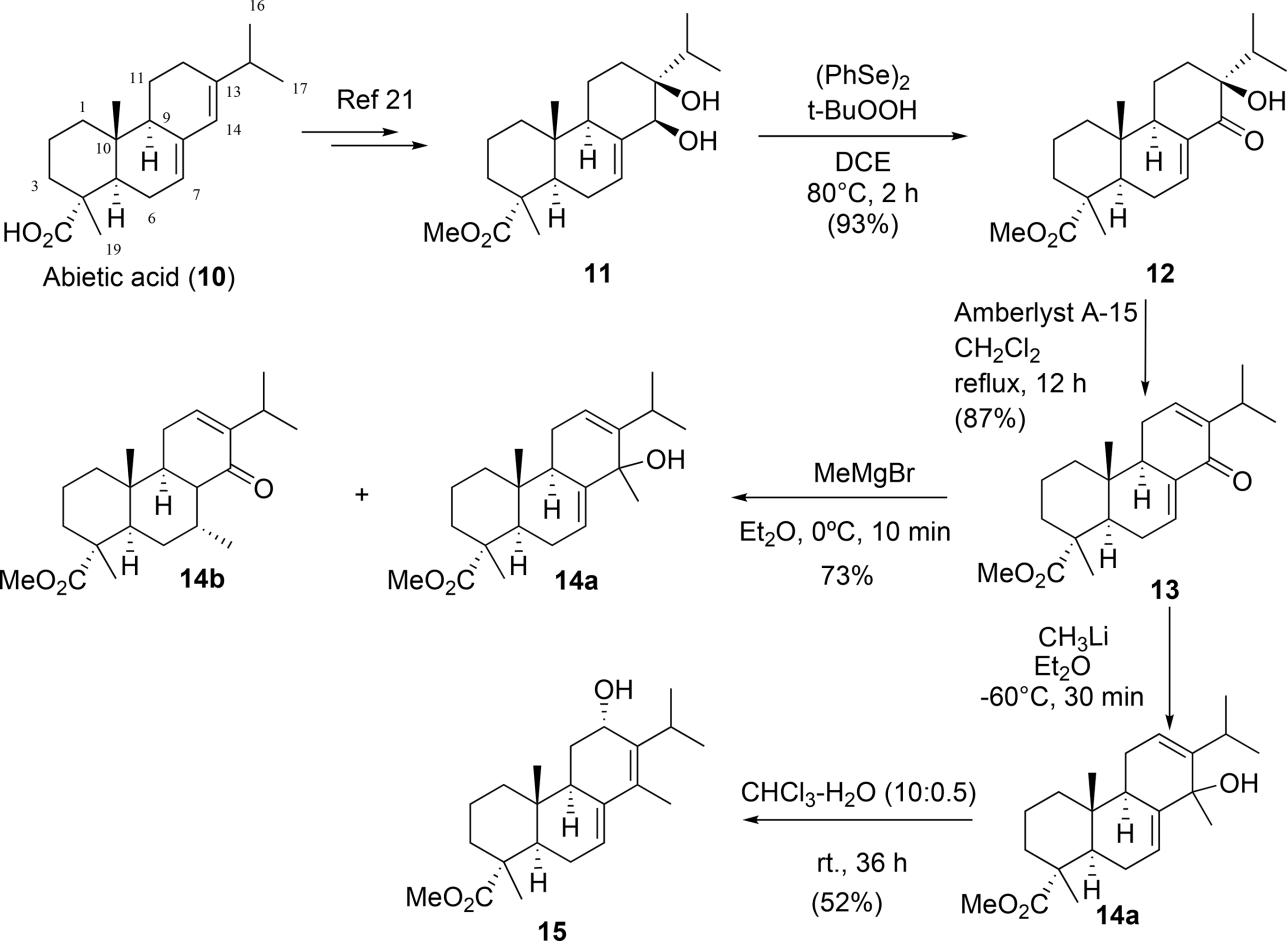
Synthesis of Dienol **15** from Abietic Acid (**10**): Introduction of the Methyl Group on C-14

The new strategy toward taepeenin F (**6**) involved the introduction of the methyl group at C-14 through a nucleophilic addition reaction to a carbonyl group, which has not been previously reported. For this, dienone **13** has been proposed as the most suitable intermediate to achieve this aim. This dienone can be easily prepared using the procedure described by our research group.^18^ The dehydration reaction of **12** has been optimized using Amberlyst A-15 refluxing in CH_2_Cl_2_, instead of using *p*-TsOH refluxing in benzene. The nucleophilic addition of the methyl group to dienone **13** has been investigated. After testing different conditions, the 1,2-addition reaction took place selectively, when dienone **10** is treated with MeLi in Et_2_O at −60 °C, affording tertiary alcohol **14a** as a 3:1 mixture of epimers Meα-C-14 and Meβ-C-14. The use of methyl magnesium bromide provides a mixture formed mainly of **14b**, as the 1,4-addition product to the Δ^7^ double bond, together with the tertiary alcohol **14a** (mixture of diastereomers in a 2.5:1 proportion), with a ratio of 4:1 (Scheme 1). We observed that **14a** is unstable, since it undergoes an uncontrolled transformation in the presence of silica gel. When a solution of tertiary alcohol **14a** in CHCl_3_–H_2_O (10:0.5) is stirred at room temperature, it is slowly transformed into dienol **15**, which has good stability.

**Scheme 2:**
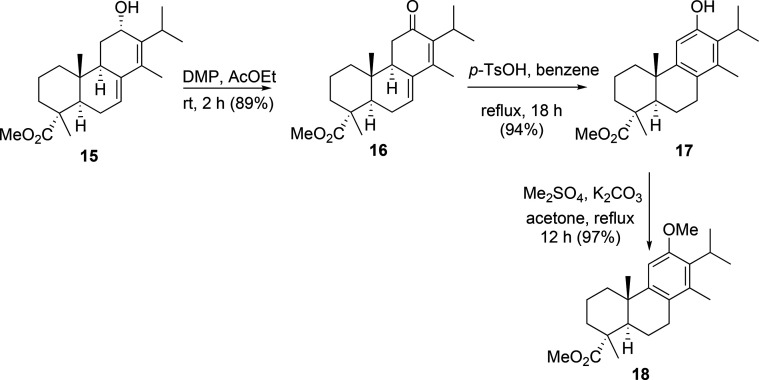
Synthesis of the Key Intermediate Methoxy Ester **18**

The next step in the synthetic sequence was to achieve the oxidation of dienol **15** to dienone **16**. This reaction was carried out efficiently with DMP in EtOAc at room temperature, affording the desired dienone **16** in good yield. This dienone was easily converted into phenol **17** by refluxing with *p*-TsOH in benzene, which was further O-methylated, to give the key intermediate **18** (Scheme 2).

**Scheme 3:**
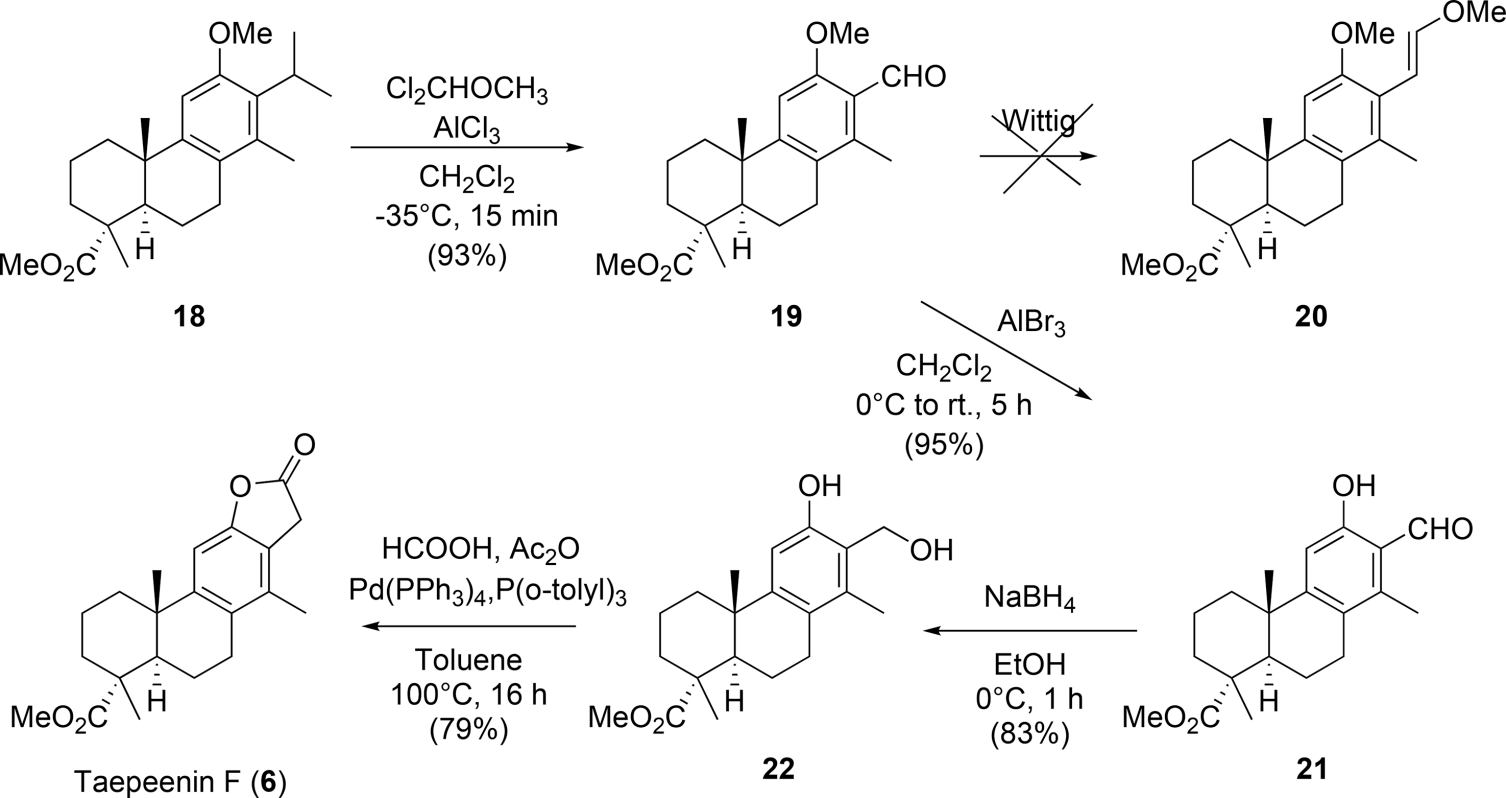
Semisynthesis of Taepeenin F (**6**) from the Methoxy Ester **18**

Finally, the compound **18** was transformed into taepeenin F (**6**). Aldehyde **19** was obtained via deisopropylation substitution of **18** by treatment with AlCl_3_, in the presence of Cl_2_CHOCH_3_ in CH_2_Cl_2_ at −35 °C. As it had been previously stated, under these mild reaction conditions, C-10 epimerization was not observed.^16^ Next, the construction of the lactone ring was investigated when **19** was treated with Ph_3_P=CHOMe; the aldehyde remained unaltered, probably due to steric effects. The alternative sequence to the synthesis of taepeenin F (**6**) from salicylaldehyde **21** was undertaken. Deprotection of the phenol group of aldehyde **19** using AlBr_3_,^19^ followed by reduction of aldehyde group, afforded hydroxy fenol **22**. The insertion of carbon monoxide in the hydroxy phenol **22**, through a Pd-catalyzed carbonylative reaction,^20^ gave the synthetic taepeenin F (**6**) (Scheme 3). The spectroscopic data of compound **6** were identical to those of the natural product.^2^

### Antiinflammatory Activity

#### Cytotoxicity on RAW 264.7 Cell Line

The growth-inhibitory effects of the compounds **6**, **16**, **17**, **21**, and **22** were analyzed on RAW 264.7 monocyte/macrophage murine cells by the well-established MTT assay.^21^ Cells were treated with gradually increased concentrations of the compound, and the viability was determined by formazan dye uptake, expressed as a percentage of untreated control cells (Figure S1 in Supporting Information).

The results showed low to moderate cytotoxic activity for all compounds (Table 1). The salicylaldehyde derivative **21** displayed the most potent effect with the lowest value of IC_50_, 21.09 μM, followed by dienone **16** exerting a moderated effect with an IC_50_ value of of 33.11 μM. Diol **22,** phenol **17**, and taepeenin F (**6**) showed weak cytotoxic effects with IC_50_ values of 101.80, 129.44, and 200.47 μM, respectively. Moreover, we have determined the IC_20_ and IC_80_ concentrations to analyze the complete range of cytotoxicity of these compounds on RAW 264.7 cells (Table 1). From above provided information, subcytotoxic concentrations corresponding to 3/4IC_50_, 1/2IC_50_, and 1/4IC_50_ were used further in the next assays, to ensure that the possible anti-inflammatory effect was exclusively due to the anti-inflammatory properties of these compounds and not cytotoxic effects.

**Table 1:** Growth-Inhibitory Effects of Tested Compounds on RAW 264.7 Monocyte/Macrophage Murine Cells

Comp. #	IC_20_ (μM)	IC_50_ (μM)	IC_80_ (μM)
**Taepeenin F**	111.40 ± 24.03	200.47 ± 4.11	254.69 ± 1.48
**16**	30.12 ± 0.49	33.11 ± 3.62	33.81 ± 0.08
**17**	27.82 ± 8.38	129.44 ± 7.06	247.44 ± 6.30
**21**	17.76 ± 1.34	21.09 ± 0.96	25.27 ± 0.58
**22**	92.08 ± 1.13	101.80 ± 0.52	114.17 ± 0.17

#### Inhibition of NO Production

**Figure 1:**
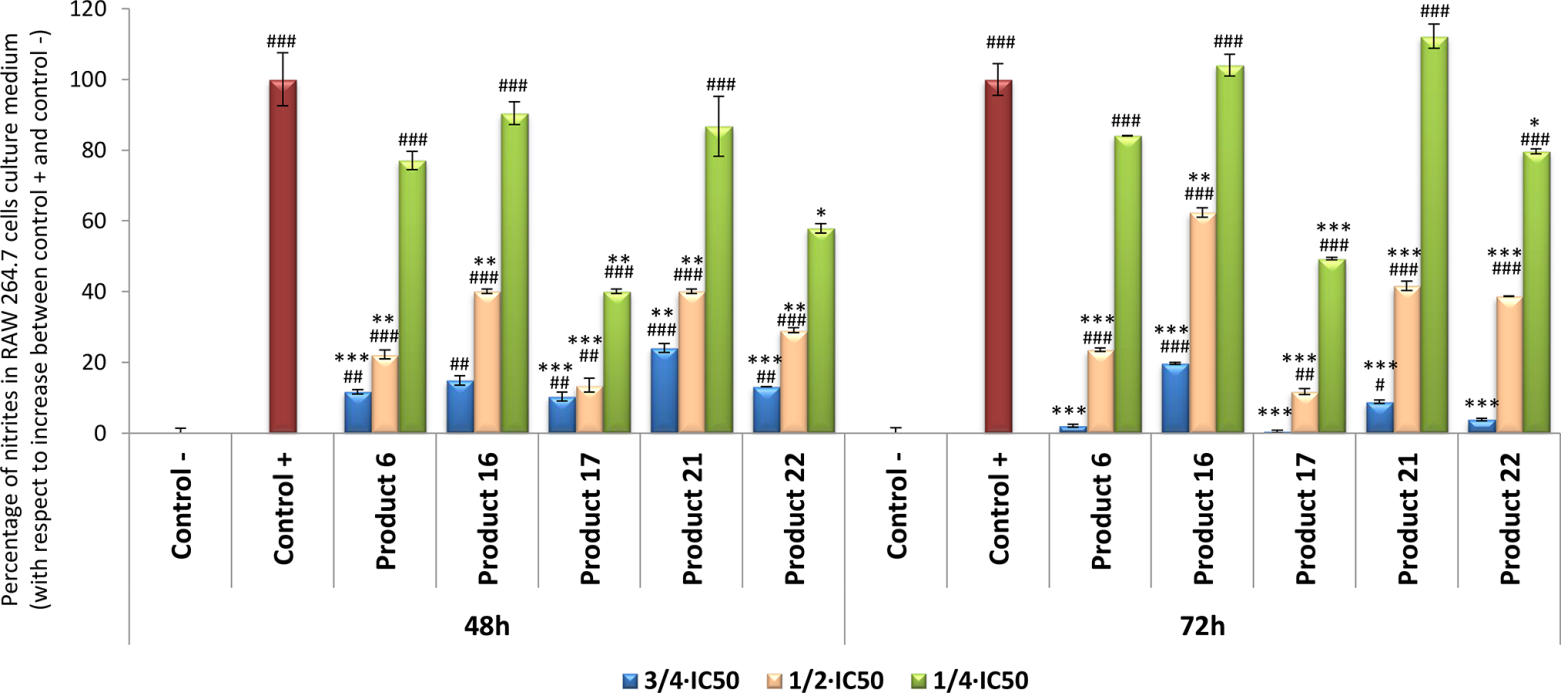
Effect of compounds **6**, **16**, **17**, **21**, and **22** on the release of nitrites assay in LPS-activated RAW 264.7 macrophage murine cells at 1/4IC_50_, 1/2IC_50_, and 3/4IC_50_ concentrations, after 48 and 72 h of treatment. Negative control (untreated cells); positive control (cells only treated with LPS); samples (cells treated with LPS and compounds). Data represent the mean ± SD of at least two independent experiments performed in triplicate. Key: *p* < 0.05 (#), *p* ≤ 0.01 (##), *p* ≤ 0.001 (###) compared to negative control; *p* < 0.05 (*), *p* ≤ 0.01 (**), *p* ≤ 0.001 (***) compared to positive control.

Macrophages activated with LPS for 24 h were incubated with compounds (**6**, **16**, **17**, **21**, and **22**) for 24, 48, and 72 h. The concentrations of nitrites were determined by the Griess reaction. The results showed that all tested compounds exhibited very important inhibition of NO release (Figure 1). After 48 h of incubation, the higher anti-inflammatory effect was induced by the phenol **17** with 89.70 and 86.5% of inhibition of NO release, respectively, to the controls, at noncytotoxic concentrations 3/4IC_50_ (97.08 μM) and 1/2IC_50_ (64.72 μM), respectively. Diterpenoids **6**, **16**, and **22** also exerted very strong inhibitory activity at 3/4IC_50_ concentration, with inhibition percentages around 85% (being 88.33, 85.13, and 86.96%, respectively). At 1/2IC_50,_ the percentages were 77.80, 59.95, and 70.94%, respectively. At 3/4IC_50_ concentration, the lowest anti-inflammatory effect was shown by **21** with an inhibition of 76.49% in NO production, which is a very significant inhibitory rate. Generally, after 72 h, the inhibition was even more pronounced than after 48 h of treatment. At this time, all compounds displayed strong inhibition of the inflammatory process, with percentages of inhibition of NO release between 80 and 99% at 3/4IC_50_ concentrations. In addition, at 1/2IC_50_ concentrations, the results were similar to the ones found after 48 h of treatment. However, the lowest values of inhibition of NO production were obtained at 1/4IC_50_ concentrations, at all times assayed.

The highest inhibition of the NO production (99%) was reached after treating with compound **17**, displaying its strongest effect after 72 h of treatment (increasing by 10% with respect to the results found after 48 h of treatment), followed by compounds **6**, **22,** and **21** with 98, 96, and 91%, to depress NO release, respectively. The lowest inhibition rate was reached using compound **21** after 48 h of treatment (76%) and with compound **16** after 72 h of treatment (80%).

#### RAW 264.7 Cell-Cycle Arrest and Distribution

Since the anti-inflammatory effect can be related to the ability to change the inflammatory processes induced by LPS in RAW 264.7 cells, including monocyte/macrophage cell differentiation and cell-cycle arrest,^22^ we analyzed the cell-cycle distribution in these cells in response to LPS and compounds **16** and **21**. The two compounds with the highest antiproliferative and important NO inhibitory effects were selected for this assay. For this purpose, flow cytometry by propidium iodide (PI) staining was used to measure DNA ploidy as well as the distribution of cells in every phase of the cell cycle (Figure S2). After 72 h of treatment with products **16** and **21**, DNA histogram analysis showed that these compounds could revert the cell-cycle arrest induced by LPS.

**Figure 2:**
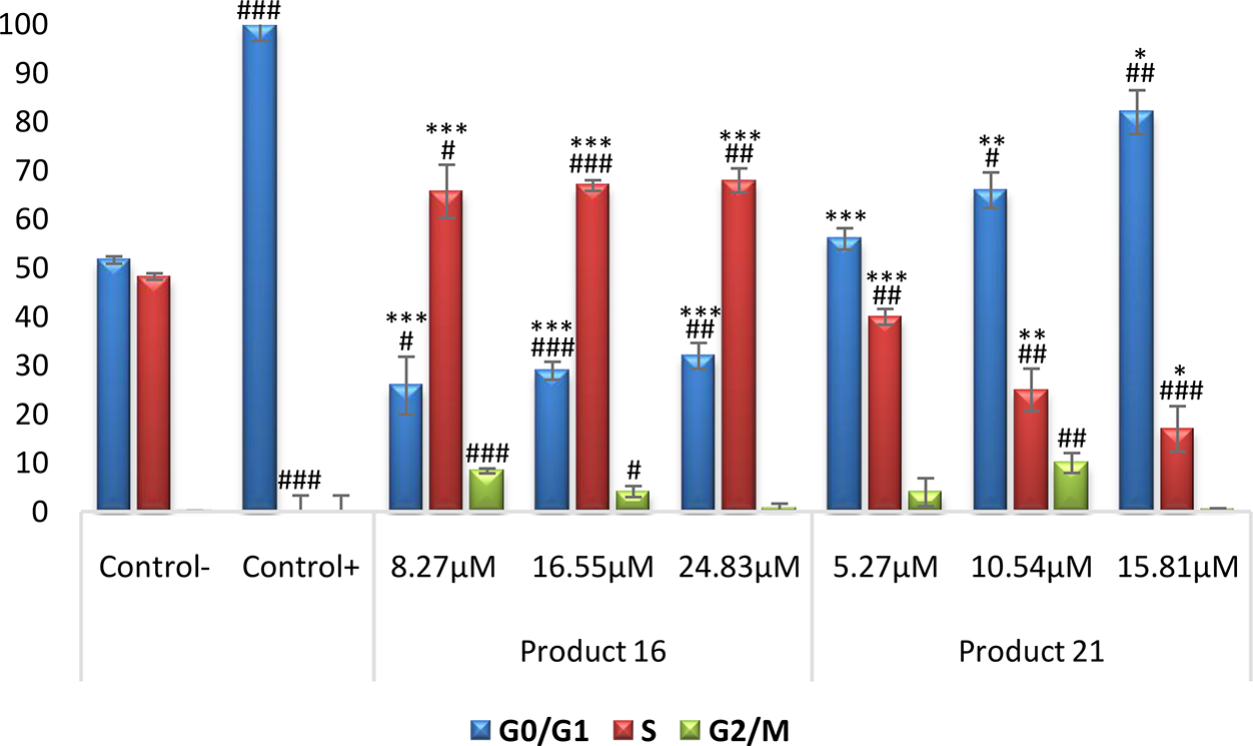
Percentage of RAW 264.7 macrophage murine cells in each cell-cycle phase after treatment with compounds **16** and **21** at their 1/4IC_50_, 1/2IC_50_, and 3/4IC_50_ concentrations for 72 h. G0/G1 phase (blue bars), S phase (red bars), and G2/M phase (green bars). Negative control (untreated cells); positive control (cells treated only with LPS); samples (cells treated with LPS and compounds **16** and **21**). Data represent the mean ± SD of at least two independent experiments performed in triplicate. Key: *p* < 0.05 (#), *p* ≤ 0.01 (##), *p* ≤ 0.001 (###) compared to negative control; *p* < 0.05 (*), *p* ≤ 0.01 (**), *p* ≤ 0.001 (***) compared to positive control.

These results showed that dienone **16** inhibited the LPS-induced differentiation of RAW 264.7 macrophages in a dose-dependent manner by increasing the proportion of cells in the S phase to 69% and decreasing the cell number in G0/G1 (between 75 and 68%) and G2/M phases (from 8 to 0.3%,) with respect to positive control cells (treated only with LPS) (Figure 2), probably as a consequence of the NO inhibitory effect induced by this compound at the concentrations assayed. Compound **21** induced similar effects, decreasing the proportion of cells in the G0/G1 phase from 44 to 19% and decreasing the cell number in the S phase from 38% to 17%, with respect to positive control cells. In this case, the changes in G2/M cells were not clearly dependent on the concentration. Therefore, we have shown that the tested compounds exert significant inhibition of NO release and inhibited the LPS-induced cell-cycle arrest in activated macrophages RAW 264.7 in a dose-dependent manner, thus showing an important immunomodulatory activity. To clarify the mechanism underlying this activity, deepened molecular assays need to be performed in further studies.

Previous reports have described the anti-inflammatory potential of natural cassane diterpenoids from different species of *Caesalpinia sp*. This activity was showed by inhibition of nitric oxide production and interleukin-1 (IL-1), interleukin-6 (IL-6) protein expression,^23,24^ and other inflammatory protein mediators, such as cyclooxygenase-2 (COX-2)^25,26^ or nuclear factor κB (NF-κB).^27,28^ Yodsaoue et al. reported the inhibition of NO release induced by pulcherrin Q, a cassane diterpenoid isolated from the roots of *C. pulcherrima*, possessing potent NO inhibitory properties, with an IC_50_ value of 2.9 μM.^29^ Liu et al. confirmed that caesalmin A and caesalpinins D and H extracted from the seeds of *C. minax* displayed a moderate NO inhibitory effect, with IC_50_ values around 25.40 μM.^30,31^

### Antiproliferative Activities

#### Cancer Cell Proliferation Assay

The five newly synthesized diterpenoids were evaluated for their cytotoxic effects against three selected tumor cell lines, B16-F10 murine melanoma cells, HT29 human colon adenocarcinoma cells, and HepG2 human hepatocarcinoma cells at increasing concentrations (0–100 μg/mL). After 72 h of treatment, cell viability was determined by an MTT assay, where the tetrazolium dye was transformed into formazan in the mitochondria of viable cells, its absorbance measured at 570 nm and expressed as a percentage with respect to untreated control cells (S3 Figure.). The concentrations required for 20, 50, and for 80% growth inhibition (IC_20_, IC_50_, and IC_80_) were also determined for each compound (Table 2).

**Table 2:** Growth-Inhibitory Effects of the Tested Compounds on the Three Cancer Cell Lines

Cell line	Comp. #	IC_20_ (μM)	IC_50_ (μM)	IC_80_ (μM)
**B16-F10**	**Taepeenin F (6)**	233.91 ± 4.12	256.82 ± 24.00	N/A
	**16**	14.17 ± 0.19	22.16 ± 1.16	35.75 ± 3.35
	**17**	33.31 ± 5.94	132.66 ± 8.17	259.30 ± 1.35
	**21**	3.18 ± 2.30	7.72 ± 3.92	20.14 ± 7.72
	**22**	85.46 ± 6.16	101.62 ± 4.36	122.47 ± 4.22
**HepG2**	**Taepeenin F (6)**	111.60 ± 21.98	236.95 ± 9.69	N/A
	**16**	5.05 ± 2.74	18.47 ± 4.73	72.41 ± 8,90
	**17**	32.65 ± 3.41	137.25 ± 13.87	273.93 ± 41.23
	**21**	33.22 ± 8.64	37.85 ± 5.40	45.27 ± 0.90
	**22**	110.11 ± 0.84	125.13 ± 2.36	140.72 ± 6.51
**HT29**	**Taepeenin F (6)**	124.25 ± 1.11	391.43 ± 39.50	N/A
	**16**	21.40 ± 3.09	33.40 ± 2.16	55.01 ± 0.93
	**17**	122.32 ± 24.25	222.34 ± 22.45	310.51 ± 8.32
	**21**	13.53 ± 0.55	20.07 ± 0.61	30.55 ± 0.81
	**22**	67.48 ± 4.61	85.61 ± 4.99	114.5 ± 7.35

All the tested compounds induced a dose-dependent decrease in the viability of cells after 72 h of treatment. The lowest IC_50_ values were observed for compound **21** (7.72 μM in B16-F10 cells, 37.85 μM in HepG2 cells, and 20.07 μM in HT29 cells) and for compound **16** (22.16 μM in B16-F10 cells, 18.47 μM in HepG2 cells, and 33.40 μM in HT29 cells). These results showed that taepeenin F (**6**) did not display relevant cytotoxic effects, with the highest IC_50_ values (IC_50_ > 100 μM) in the three cancer-cell lines. There are no available data on the biological activity of taepeenin F (**6**) until now. Compounds **17** and **22** also were inactive in the three assayed cell lines with IC_50_ > 85.61 μM.

Consequently, we have selected the salicylaldehyde derivative **21** and dienone **16** to further investigate their antitumor activity in B16-F10 cells, using cytometric assays to determine their effects on the cell cycle, apoptosis characterization, and changes in mitochondrial membrane potential.

#### B16-F10 Cell-Cycle Arrest and Distribution

**Figure 3:**
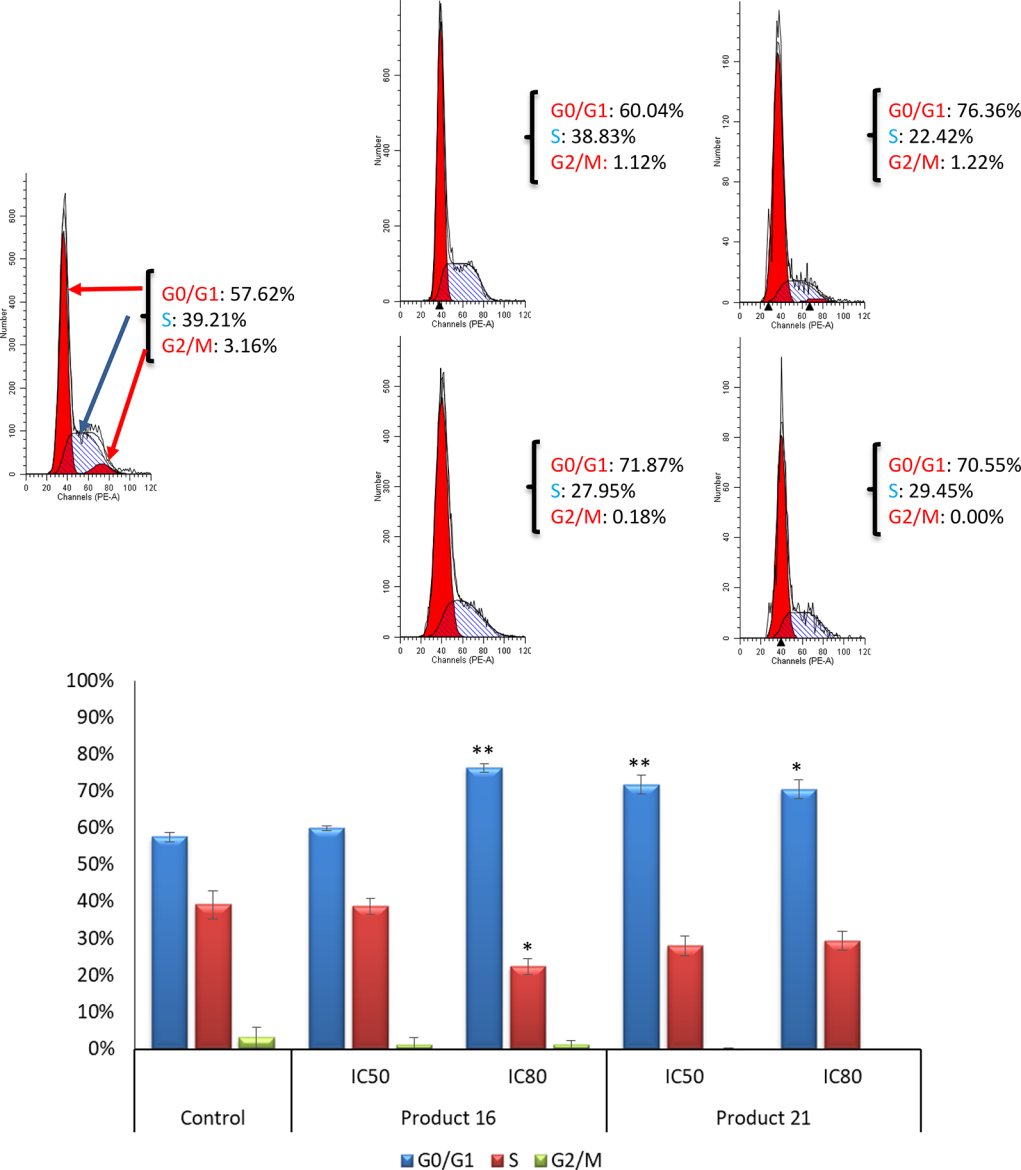
Top: Histograms of cell cycle of B16-F10 skin-melanoma cells, after 72 h of treatment with compounds **16** and **21**, at IC_50_ and IC_80_ concentrations. G0/G1 phase (red), S phase (blue), and G2/M phase (red). Bottom: Percentage of cells in each cell-cycle phase: G0/G1 phase (blue bars), S phase (red bars), and G2/M phase (green bars). Control (untreated cells); samples (cells treated with compounds **16** and **21**). Each value represents mean ± SD of at least three independent experiments performed in duplicate. Key: *p* < 0.05 (*), *p* ≤ 0.01 (**), and *p* ≤ 0.001 (***) with respect to untreated control cells.

The effects of the compounds **16** and **21** on the B16-F10 cell cycle were investigated using flow cytometry through propidium iodide staining. With this assay, we can determine a possible cytostatic effect related to the cytotoxic response and also measure DNA ploidy as well as alterations of cell-cycle profiles.^32^ The distribution of cells in different cell-cycle phases was analyzed by the incorporation of propidium iodide (PI), after treatment of B16-F10 cells with the products **16** and **21** for 72 h at IC_50_ and IC_80_ concentrations, previously determined. The results obtained in this analysis are shown in Figure 3. Compounds **16** and **21** significantly arrested the cell cycle, increasing the number of cells in the G0/G1 phase. The dienone **16** had the greatest effect, reaching 76.36% of cells in the G0/G1 phase at IC_80_ concentration, an increase of 18.24% over the untreated control cells. Nevertheless, at the IC_50_ concentration, this product had no significant effect, probably due to its apoptotic and cytotoxic effects. The aldehyde **21** induced the cell-cycle arrest in the G0/G1 phase at both IC_50_ and IC_80_ concentrations, with values of 71.87 and 70.55%, respectively, representing increases of 13.75 and 12.43% with respect to nontreated control cells, respectively. These increases were accompanied by a concomitant decrease of the percentage of cells in the S phase, with a percentage of 22.42% at the IC_80_ concentration for the product **16** (a decrease of 16.79% compared to untreated control cells). In the case of product **21**, the rates of the cell population in the S phase were 27.95 and 29.45% for IC_50_ and IC_80_ concentrations (decreasing only by 11.26 and 9.76% with respect to untreated control cells), respectively. Lower changes were observed in the G2/M cell-cycle phase (Figure 3).

#### Characterization of Apoptotic Effects by Flow Cytometry with Annexin V

After treating B16-F10 cells with compounds **16** and **21** at the IC_50_ and IC_80_ concentrations for 24, 48, and 72 h, the determination of the apoptotic cell percentage was conducted through double staining with annexin V, conjugated fluorescein isothiocyanate (FITC), and propidium iodide (PI). Cytometric FACS analysis using annexin V-FITC and PI staining was used to differentiate four cell populations (Figure 5): normal cells (annexin V^–^ and PI^–^), early apoptotic cells (annexin V^+^ and PI^–^), late apoptotic cells (annexin V^+^ and PI^+^), and necrotic cells (annexin V^–^ and PI^+^).^33^

Compounds **16** and **21** showed significant apoptotic effects on B16-F10 cells, with high total apoptosis percentages. After 24 h of treatment, compounds **16** and **21** did not exert a significant effect. Nevertheless, after 48 h of treatment at the IC_80_ concentration, the total apoptosis percentages reached 26.6% (12.06% early apoptosis and 14.53% late apoptosis) for compound **16** and 32.86% (5.73% early apoptosis and 27.13% late apoptosis) for compound **21**. At the IC_50_ concentration, these percentages were 13.13% (3.73% early and 9.40% late apoptosis) for compound **16** and 25.23% (6.93% early apoptosis and 18.30% late apoptosis) for compound **21**.

The highest percentage of apoptosis was observed at the IC_80_ concentration after 72 h of treatment. At this concentration, the percentages of total apoptosis were 48.76% (27.63% early apoptosis and 21.13% late apoptosis) in response to compound **16** and 41.23% (23.56% early apoptosis and 17.66% late apoptosis) for compound **21**. At the IC_50_ concentration, these percentages reached 22.36% (5.33% early apoptosis and 17.03% late apoptosis) for compound **21** and only 7.7% (0.06% early and 7.63% late apoptosis) for compound **16** (possibly its high cytotoxicity caused the disappearance of this cell population at this time). In addition, the percentages of the necrotic population were unnoticeable, at the used concentrations and times during this experiment.

**Figure 4:**
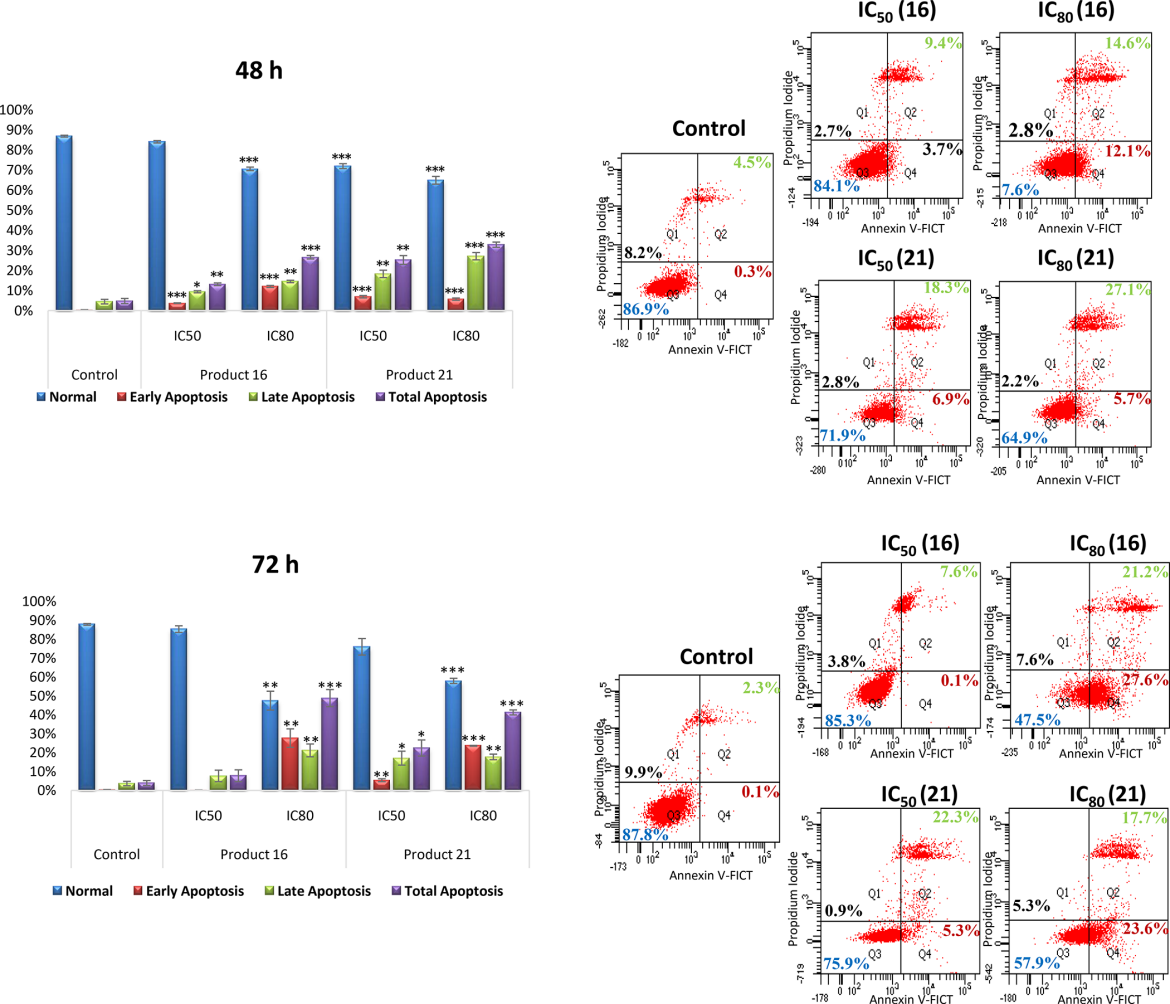
Effects of compounds **16** and **21** on apoptosis in B16-F10 skin-melanoma cells, after 48 and 72 h of treatment, at IC_50_ and IC_80_ concentrations. Right: Diagrams of annexin V/propidium iodide (IP) cytometry. Q1, necrotic cells (annexin V^–^ PI^+^); Q2, late apoptotic cells (annexin V^+^ PI^+^); Q3, normal cells (annexin V^–^ PI^–^); Q4, early apoptotic cells (annexin V^+^ PI^–^). Control (untreated cells), samples (cells treated with compounds **16** and **21**). Left: Flow cytometry analysis of annexin V-FITC staining and PI accumulation: normal cells (blue bars), early apoptotic cells (red bars), late apoptotic cells (green bars), total apoptotic cells (purple bars). Each value represents mean ± SD of three experiments in duplicate. Key: *p* < 0.05 (*), *p* ≤ 0.01 (**), and *p* ≤ 0.001 (***) with respect to untreated control cells.

The results showed an increase in the apoptotic population cells in a concentration-dependent manner. The late apoptosis population of cells was higher than the one of the initial apoptosis. The high percentages of apoptosis attained by these derivatives indicate that they exert their cytotoxic action by apoptosis induction and could be used as promising anticancer agents. The identification of new cytotoxic elements that enhance or restore the capability of malignant tumor cells to undergo apoptosis may be crucial for more effective anticancer therapies.^34^

#### Mitochondrial Membrane Potential Disturbances

**Figure 5:**
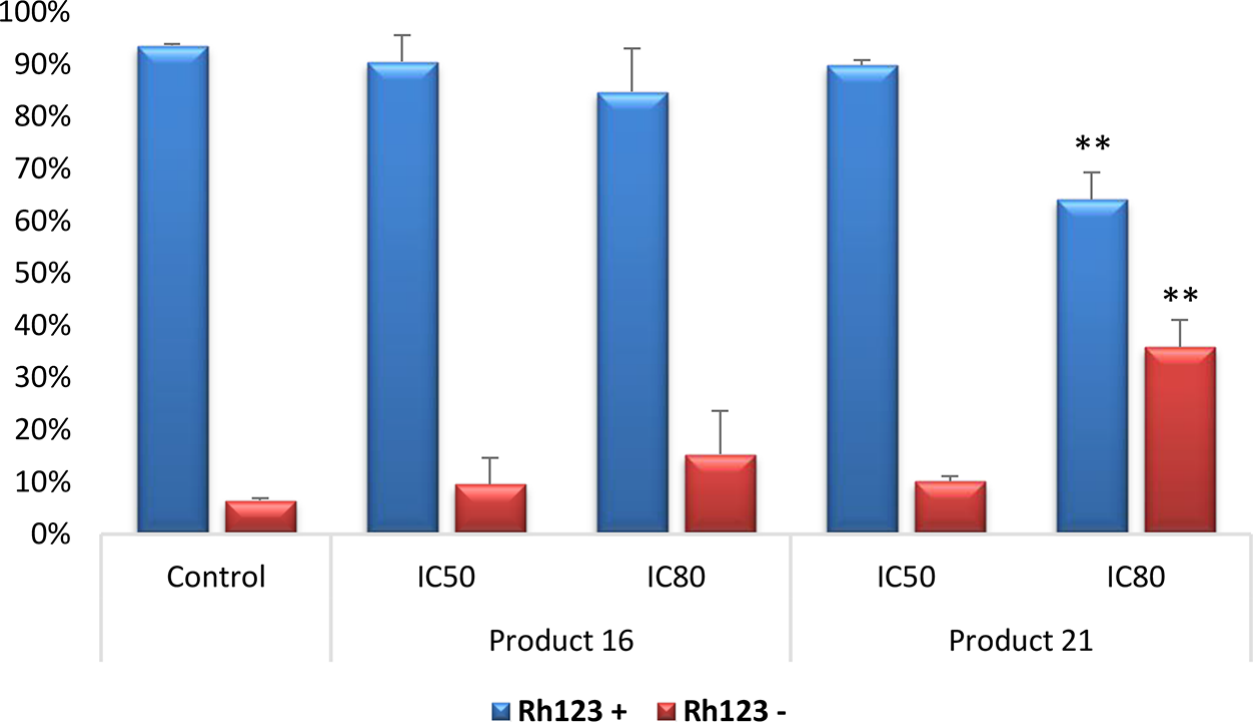
Percentages of B16-F10 Rh123 positive and Rh123 negative cells after 72 h of treatment with compounds **16** and **21** at IC_50_ and IC_80_ concentrations. Control (untreated cells), samples (cells treated with compounds **16** and **21**). Each value is expressed as mean ± SD of two independent experiments, performed in triplicate. Key: *p* < 0.05 (*), *p* ≤ 0.01 (**), and *p* ≤ 0.001 (***) with respect to untreated control cell.

Changes in MMP were analyzed by flow cytometry staining with Rh123/IP (Figure 5), after treatment with the compounds **16** and **21** for 72 h, at IC_50_ and IC_80_ concentrations, to determine the possible mechanism involved in the apoptotic responses, in the B16-F10 cell line. Treatment with the dienone **16** did not produce any changes in MMP, which suggests the activation of the extrinsic apoptotic pathway, since at this time and these concentrations this product is clearly apoptotic. Salicylaldehyde **21** showed similar results; nevertheless, at the IC_80_ concentration, the population of Rh123 negative cells was higher that the one found in the control untreated cells, probably due to the high cytotoxicity of compound **21** at this time and concentration. In addition, the loss of part of MMP can be related to the secondary activation of the intrinsic apoptotic pathway as a final event in the extrinsic apoptosis mechanism. Further molecular studies will be necessary to confirm these conclusions.

There are few studies on the cytotoxicity and anticancer potential of cassane-type diterpenoids. Recent studies have shown that cassane-type diterpenes displayed cytotoxic and antineoplastic activities against various tumor cell lines,^25,26,28^

## Conclusion

We have reported a new strategy toward the preparation of aromatic cassane-type diterpene taepeenin F (**6**) from abietic acid (**10**). Antiproliferative and NO inhibitory activities have been evaluated for compounds: **6**, **16**, **17**, **21**, and **22**. The antiproliferative effects were tested in vitro against different types of cancer cell lines. The highest cytotoxic effect was induced by compounds **16** and **21**. The antiproliferative effect induced by compounds **16** and **21** involved inducing apoptosis-mediated G0/G1 cell-cycle arrest in B16-F10 murine melanoma cells. The mitochondrial membrane potential analysis showed that the apoptosis induction by the dienone **16** seems to be initiated by the activation of the extrinsic apoptotic pathway. In the case of the aldehyde **21,** the MMP results suggest the activation of the extrinsic pathway followed by an intrinsic one as the final event in the apoptotic mechanism. All tested compounds showed a very potent inhibition effect of the NO production ≥89% in LPS-activated RAW 264.7 murine monocyte/macrophage, at the subcytotoxic concentrations (1/4IC_50_, 1/2IC_50_, and 3/4IC_50_), ensuring that this inhibition is not due to their cytotoxic effect. Compounds **16** and **21** could revert the 100% cell-cycle arrest in the G0/G1 phase induced by LPS in RAW 264.7 cells, activating cell proliferation and cell-cycle progression. The results indicate that compounds **16** and **21** should be further investigated as potential antitumor and anti-inflammatory agents. Further studies are necessary to elucidate the cellular and molecular elements involved in their bioactivities, and the levels of activity/toxicity should be evaluated in preclinical models.

## Experimental Section

### Chemistry

#### General Experimental Procedures

Unless stated otherwise, reactions were performed in oven-dried glassware under an argon atmosphere using dry solvents. Solvents were dried as follows: benzene over Na and benzophenone, dichloromethane (DCM) over CaH_2_. Thin-layer chromatography (TLC) was performed using F254 precoated plates (0.25 mm) and visualized by UV fluorescence quenching and phosphomolybdic acid solution staining. Flash chromatography was performed on silica gel (230–400 mesh). Chromatography separations were carried out by a conventional column on silica gel 60 (230–400 mesh), using hexane–ethyl acetate (AcOEt/hexane) mixtures of increasing polarity. ^1^H and ^13^C NMR spectra were recorded at 500 and 400 MHz and at 125 and 100 MHz, respectively. Chemical shifts (δ_Η_) are quoted in parts per million (ppm) referenced to the appropriate residual solvent peak and tetramethylsilane. Data for ^1^H NMR spectra are reported as follows: chemical shift (δ ppm) (multiplicity, coupling constant (Hz), integration), with the abbreviations s, br s, d, br d, dd, hept, and m denoting singlet, broad singlet, doublet, broad doublet, triplet, double doublet, heptuplet, and multiplet, respectively. *J* = coupling constant in Hertz (Hz). Data for ^13^C NMR spectra are reported in terms of chemical shift relative to Me_4_Si (δ_C_ 0.0), and the signals were assigned utilizing DEPT experiments and on the basis of heteronuclear correlations. Infrared spectra (IR) were recorded as thin films or as solids on a FTIR spectrophotometer with samples between sodium chloride plates and are reported in frequency of absorption (cm^–1^). Only selected absorbances (ν_max_) are reported. ([α]_D_^25^) measurements were carried out in a polarimeter, utilizing a 1 dm length cell and CHCl_3_ as a solvent. Concentration is expressed in mg/mL. HRMS were recorded on a spectrometer, utilizing a Q-TOF analyzer, and ESI^+^ ionization.

#### Synthesis

##### (1R,4aR,7S,10aR)-Methyl 7-hydroxy-7-isopropyl-1,4a-dimethyl-8-oxo-1,2,3,4,4a,4b,5,6,7,8,10,10a-dodecahydrophenanthrene-1-carboxylate (**12**)

To a stirred solution of **11** (8.48 g, 24.2 mmol) in 1,2-dichloroethane (90 mL) were added diphenyl diselenide (8.6 g, 27.6 mmol) and *t*-BuO_2_H (7.3 mL, 36.4 mmol, 5 M in decane), and the mixture was stirred at 80 °C under argon atmosphere for 2 h, at which time, TLC showed no remaining starting material. Then, the solvent was evaporated, and the residue was purified by flash chromatography on silica gel (20% AcOEt/hexane) affording compound **12** (7.83 g, 93%) as a white solid.

##### (1R,4aR,10aR)-Methyl 7-isopropyl-1,4a-dimethyl-8-oxo-1,2,3,4,4a,4b,5,8,10,10a-decahydrophenanthrene-1-carboxylate (**13**)

To a solution of hydroxy ketone **12** (7.44 g, 21.37 mmol) in dry dichloromethane (80 mL), was added Amberlyst A-15 (3 g), and the mixture was heated at reflux for 12 h, at which time TLC showed the disappearance of starting material. Then, the reaction mixture was filtrated, and the solvent was evaporated to give a crude product, which was purified by flash chromatography on silica gel (15% AcOEt/hexane) affording compound **13** (6.13 g, 87%) as a yellow syrup.

The spectroscopic data of compounds **12** and **13** were in agreement with those described in the literature.^19^

##### Treatment of Dienone **13** with CH_3_Li

To a solution of dienone **13** (320 mg, 0.97 mmol) in anhydrous diethyl ether (10 mL) cooled to −60 °C and under argon atmosphere, methyllithium (0.4 mL, 1.2 mmol, 3 M in diethoxymethane) was slowly added. The reaction mixture was stirred at −60 °C for 30 min, at which time TLC showed no starting material. Then, Et_2_O–H_2_O (15:10 mL) was slowly added, and the mixture was stirred at room temperature for 10 min. Next, the phases were shaken and separated; the organic phase was washed with brine and dried over anhydrous Na_2_SO_4_. Removal of the solvent under vacuum afforded 309 mg of compound **14a**, as a 3:1 mixture of epimers Meα-C-14 and Meβ-C-14. **14a** was used in the next step without purification. ^1^H NMR (400 MHz, chloroform-*d*) δ (ppm) signals assignable to the major isomer: 0.75 (s, 3H), 0.87 (d, *J* = 6.8 Hz, 3H), 0.90 (d, *J* = 6.8 Hz, 3H), 1.22 (s, 3H), 1.26 (s, 3H), 1.88 (hept, *J* = 6.9 Hz, 1H), 2, 65 (m, 1H), 2.67 (br s, 1H), 3.66 (s, 3H), 5.34 (br d, *J* = 10.0 Hz, 1H), 6.85 (dd, *J* = 10.0, 3.2 Hz, 1H). Signals assignable to the minor isomer: 0.79 (s, 3H), 0.91 (d, *J* = 7.0 Hz, 3H), 0.96 (d, *J* = 7.0 Hz, 3H), 1.25 (s, 3H), 1.29 (s, 3H), 3.66 (s, 3H), 5.55 (dd, *J* = 9.7, 3.0 Hz, 1H), 6.21(dd, *J* = 9.8, 3.1 Hz, 1H). ^13^C NMR (100 MHz, chloroform-*d*) δ (ppm) signals assignable to the major isomer 179.0 (C), 142.2 (C), 134.6 (C), 130.2 (CH), 125.1 (CH), 77.9 (C), 60.7 (CH), 52.1 (CH_3_), 50.72 (CH), 46.2 (C), 38.0 (C), 37.1(CH_3_), 37.06 (CH_2_), 36.3 (CH_2_), 34.8 (CH_2_), 26.0 (CH), 21.8 (CH_2_), 18.0 (CH_2_), 17.6 (CH_3_), 17.4 (CH_3_), 16.9 (CH_3_), 13.5 (CH_3_). IR (film): 3531, 2947, 2869, 1726, 1457, 1384, 1242, 1150 cm^–1^. HRMS (ESI) *m*/*z*: calcd for C_22_H_34_O_3_ Na (M+Na+) 369.2406, found: 369.2403.

##### Treatment of Dienone **13** with Methyl Magnesium Bromide

A solution of methyl magnesium bromide (1 mL, 1.4 mmol, 1.4 M) was added to a solution of dienone **13** (0.4 g, 1.21 mmol) in dry ethyl ether (10 mL), previously cooled to 0 °C under an argon atmosphere. The reaction mixture was stirred for 10 min, at which time TLC showed the disappearance of compound **13**. Then, water (3 mL) was added, and the mixture was extracted with ethyl acetate (2 × 10 mL). The organic phase was dried over anhydrous Na_2_SO_4_, filtered, and evaporated under reduced pressure, obtaining 387 mg of a mixture of **14a** and **14b** (ratio 1:4). The crude product was purified by flash chromatograph, using 10% AcOEt/hexane, to give compounds **14b** (305 mg, 73%) and **14a** (13 mg, 3%). (1R,4aR,4bR,10aR)-Methyl 7-isopropyl-1,4a-dimethyl-8-oxo-1,2,3,4,4a,4b, 5,8,10,10a-decahydrophenantrene-1-carboxylate (**14b**). [α]_D_^25^ = −45.14 (c 0.14, CHCl_3_). ^1^H NMR (500 MHz, chloroform-d) δ (ppm) 0.78(d, *J* = 6.8 Hz, 3H), 0.85 (s, 3H), 0.89 (d, *J* = 7.0 Hz, 3H), 0.93 (d, *J* = 6.8 Hz, 3H), 1.04 (m,1 H), 1.10 (s, 3H), 1.18–1.27 (m, 3H), 1.51–2.65 (m, 10H), 2.79 (m, 1H), 3.57 (s, 3H), 6.57 (d, *J* = 6.9 Hz, 1H). ^13^C NMR (125 MHz, CDCl_3_) δ 206.94 (C=O), 200.31 (C=O), 178.93 (C), 145.41(C), 140.89 (CH), 51.80 (CH), 49.35 (CH), 47.33 (C), 45.72 (CH), 42.00 (CH), 37.70 (CH_2_), 36.85 (C), 36.77 (CH_2_), 31.12 (CH_2_), 30.93 (CH), 28.42 (CH_3_), 26.26 (CH_3_), 23.61(CH_2_), 22.40 (CH_3_), 21.25 (CH_3_), 18.00 (CH_2_), 16.59 (CH_3_), 14.15 (C), 14.10 (CH_3_). IR (film): 610, 635, 693, 754, 959, 1075, 1124, 1254, 1287, 1425, 1721, 2912, 2958, 2924 cm^–1^.

##### Treatment of Dienol **14a** with CHCl_3_: Synthesis of Compound **15**

A solution of crude product of **14a** (309 mg) in CHCl_3_–H_2_O (20:1 mL) was stirred at room temperature for 36 h. Then, the organic layer was separated and dried over anhydrous Na_2_SO_4_. Removal of the solvent under vacuum afforded a crude product that was purified by silica gel flash chromatography, using 10% AcOEt/hexane, to give compound **15** (174 mg, 52%) as a colorless syrup. (1R,4aR,10aR)-Methyl 6-hydroxy-7-isopropyl-1,4a,8-trimethyl-1,2,3,4,4a,4b,5,6,10,10a-decahydrophenanthrene-1-carboxylate (**15**). [α]_D_^25^ = −94.5 (c 1.3, CHCl_3_). ^1^H NMR (500 MHz, chloroform-*d*) δ (ppm) 0.79 (s, 3H), 1.02 (d, *J* = 6.9 Hz, 3H), 1.14 (d, *J* = 6.9 Hz, 3H), 1.25 (s, 3H), 1.26–1.31 (m, 2H), 1.54–1.66 (m, 4H), 1.79 (s, 3H), 1.81–1.90 (m, 3H), 2.08–2.15 (m, 2H), 2.38 (br d, *J* = 13.7 Hz, 1H), 2.97 (hept, *J* = 6.9 Hz, 1H), 3.64 (s, 3H), 4.38 (br s, 1H), 5.77 (br s, 1H). ^13^C NMR (125 MHz, chloroform-*d*) δ (ppm) 13.6 (CH_3_), 14.5 (CH_3_), 16.9 (CH_3_), 18.2 (CH_2_), 21.5 (CH_3_), 22.0 (CH_3_), 26.3 (CH_2_), 30.2 (CH), 31.0 (CH_2_), 34.4 (C), 37.2 (CH_2_), 38.1 (CH_2_), 44.0 (CH), 44.6 (CH), 46.5 (C), 52.0 (CH_3_), 64.2 (CH), 121.3 (CH), 129.1 (C), 135.6 (C), 139.3 (C), 179.1 (C). IR (film): 3419, 2926, 1726.5, 1459, 1433, 1385, 1243, 1151, 1104, 1144, 1024, 771 cm^–1^. HRMS (ESI) *m*/*z*: calcd for C_22_H_34_O_3_ Na (M+Na+) 369.2406, found: 369.2397.

##### (1R,4aR,10aR)-Methyl 7-isopropyl-1,4a,8-trimethyl-6-oxo-1,2,3,4,4a,4b,5,6,10,10a-decahydrophenanthrene-1-carboxylate (**16**)

To a solution of dienol **15** (2.58 g, 7,44 mmol) in ethyl acetate (80 mL) at room temperature, Dess-Martin periodinane (3.80 g, 8.94 mmol) was added. After 2 h, TLC showed no starting material. Then, the reaction was quenched with water, and the mixture was washed with a saturated aqueous NaHCO_3_ (10 mL) and Na_2_S_2_O_3_ (10%) solution (2 × 10 mL) and brine (20 mL). The organic layer was dried over anhydrous Na_2_SO_4_ and concentrated under reduced pressure. The crude product was purified by flash chromatography on silica gel (10% AcOEt/hexane) to obtain the dienone **7** (2.28 g, 89%) as a yellow solid; mp 100–102 °C. [α]_D_^25^ = −44.5 (c 1.0, CHCl_3_). ^1^H NMR (500 MHz, chloroform-*d*) δ (ppm) 0.85 (s, 3H), 1.15 (d, *J* = 7.0 Hz, 3H), 1.22 (d, *J* = 7.0 Hz, 3H), 1.27 (s, 3H), 1.48–1.83 (m, 7H), 1.94 (m, 1H), 2.02 (s, 3H), 2.09–2.23 (m, 2H), 2.32–2.46 (m, 2H), 3.08 (hept, *J* = 7.0 Hz, 1H), 3.66 (s, 3H), 6.20 (br s, 1H). ^13^C NMR (125 MHz, chloroform-*d*) δ(ppm) 14.7 (CH_3_), 15.7 (CH_3_), 17.1 (CH_3_), 18.0 (CH_2_), 20.3 (CH_3_), 21.5 (CH_3_), 26.6 (CH_2_), 28.1 (CH), 35.1 (C), 37.1 (CH_2_), 38.0 (CH_2_), 38.7 (CH_2_), 43.4 (CH), 46.2 (C), 49.6 (CH), 52.2 (CH_3_), 128.4 (CH), 135.4 (C), 139.9 (C), 147.2 (C), 178.9 (C), 200.0 (C). IR (film): 2926, 1727, 1666, 1387, 1247, 1148, 772 cm^–1^. HRMS (ESI) *m*/*z*: calcd for C_22_H_33_O_3_ (M+H+) 345.2430, found: 345.2431.

##### (1R,4aS,10aR)-Methyl 6-hydroxy-7-isopropyl-1,4a,8-trimethyl-1,2,3,4,4a,9,10,10a-octahydrophenanthrene-1-carboxylate (**17**)

To a solution of dienone **16** (0.6 g, 1.74 mmol) in anhydrous benzene (20 mL) was added *p*-toluenesulfonic acid (1,25 g, 6.57 mmol), and the reaction mixture was stirred at reflux for 18 h. The mixture was filtered through silica gel to give phenol **17** (562 mg, 94%) as a white solid; mp 197–200 °C. [α]_D_^25^ = +52.5 (c 1.3, CHCl_3_). ^1^H NMR (500 MHz, chloroform-*d*) δ (ppm) 1.21 (s, 3H), 1.26 (s, 3H), 1.35 (t, *J* = 7.0 Hz, 6H), 1.39–1.51 (m, 2H), 1.58–1.85 (m, 5H), 2.15 (s, 3H), 2.16–2.22 (m, 2H), 2.62 (ddd, *J* = 16.8, 11.4, 7.6 Hz, 1H), 2.71 (dd, *J* = 16.8, 6.7 Hz, 1H), 3.39 (hept, *J* = 7.0 Hz, 1H), 3.67 (s, 3H), 4.56 (s, 1H), 6.50 (s, 1H). ^13^C NMR (125 MHz, chloroform-*d*) δ (ppm) 15.7 (CH_3_), 16.6 (CH_3_), 18.8 (CH_2_), 20.9 (CH_3_), 21.1 (CH_3_), 22.2 (CH_2_), 25.2 (CH_3_), 28.0 (CH), 28.8 (CH_2_), 36.6 (CH_2_), 37.2 (C), 38.5 (CH_2_), 44.2 (CH), 47.7 (C), 52.1 (CH_3_), 110.0 (CH), 125.9 (C), 129.9 (C), 135.2 (C), 148.1 (C), 152.5 (C), 179.4 (C). IR (film): 3424, 2931, 1694, 1297, 1254, 1134 cm^–1^. HRMS (ESI) *m*/*z*: calcd for C_22_H_32_O_3_ (M+H+) 345.2430, found: 345.2422.

##### (1R,4aS,10aR)-Methyl 7-isopropyl-6-methoxy-1,4a,8-trimethyl-1,2,3,4,4a,9,10,10a-octahydrophenanthrene-1-carboxylate (**18**)

To a solution of phenol **17** (714 mg, 2.07 mmol) in acetone (20 mL) were added K_2_CO_3_ (485 mg, 3.51 mmol) and Me_2_SO_4_ (0.35 mL, 3.69 mmol), and the mixture was refluxed for 12 h, at which time TLC indicated no starting material. Then, the solvent was evaporated under reduced pressure; the residue was diluted with ether (60 mL) and washed with water (3 × 15 mL) and brine (15 mL). The organic phase was dried over anhydrous Na_2_SO_4_ and evaporated under vacuum to afford a crude, which was purified by flash chromatography on silica gel (5% AcOEt/hexane) to give compound **18** (719 mg, 97%) as a colorless syrup. [α]_D_^25^ = +51.2 (c 2.6, CHCl_3_). ^1^H NMR (400 MHz, chloroform-*d*) δ (ppm) 1.26 (s, 3H), 1.29 (s, 3H), 1.31 (d, *J* = 7.0 Hz, 3H), 1.32 (d, *J* = 7.0 Hz, 3H), 1.43–1.58 (m, 2H), 1.61–1.90 (m, 5H), 2.17 (s, 3H), 2.24 (m, 1H), 2.28 (m, 1H), 2.64 (ddd, *J* = 16.9, 11.2, 7.5 Hz, 1H), 2.74 (dd, *J* = 16.9, 6.8 Hz, 1H), 3.41 (m, 1H), 3.68 (s, 3H), 3.79 (s, 3H), 6.69 (s, 1H). ^13^C NMR (100 MHz, chloroform-*d*) δ (ppm) 15.70 (CH_3_), 16.62 (CH_3_), 18.85 (CH_2_), 21.03 (CH_3_), 21.15 (CH_3_), 22.19 (CH_2_), 25.18 (CH), 25.19 (CH_3_), 28.77 (CH_2_), 36.63 (CH_2_), 37.57 (C), 38.57 (CH_2_), 44.36 (CH), 47.78 (C), 52.01 (CH_3_), 55.54 (CH_3_), 105.78 (CH), 125.96 (C), 132.34 (C), 134.80 (C), 147.62 (C), 156.94 (C), 179.26 (C). IR (film): 2930, 1726, 1456, 1246, 1133, 1103 cm^–1^. HRMS (ESI) *m*/*z*: calcd for C_23_H_35_O_3_ (M+H+) 359.2586, found: 359.2581.

##### (1R,4aS)-Methyl 7-formyl-6-methoxy-1,4a,8-trimethyl-1,2,3,4,4a,9,10,10a-octahydrophenanthrene-1-carboxylate (**19**)

To a solution of compound **18** (216 mg, 0.63 mmol) in anhydrous dichloromethane (10 mL) cooled to −35 °C was added dichloromethyl methyl ether (0.16 mL, 1.81 mmol) under an argon atmosphere. To this vigorously stirred mixture, AlCl_3_ (245 mg, 1.85 mmol) was added in small portions, and the reaction mixture was stirred at −35 °C for 15 min, at which time TLC showed no starting material. The mixture was slowly poured into an ice-cold bath and extracted with ethyl acetate (2 × 15 mL). The combined organic layers were washed with water (3 × 10 mL) and brine (10 mL), dried over anhydrous Na_2_SO_4_, and concentrated under reduced pressure. The crude product was purified by flash chromatography on silica gel (5% AcOEt/hexane) to yield aldehyde **19** (201 mg, 93%) as a colorless syrup. [α]_D_^25^ = +31.8 (c 0.8, CHCl_3_). ^1^H NMR (500 MHz, chloroform-*d*) δ (ppm) 1.24 (s, 3H), 1.28 (s, 3H), 1.47–1.56 (m, 2H), 1.63–1.84 (m, 5H), 2.17 (dd, *J* = 12.7, 2.1 Hz, 1H), 2.29 (m, 1H), 2.42 (s, 3H), 2.63 (ddd, *J* = 17.1, 11.4, 7.8 Hz, 1H), 2.74 (dd, *J* = 17.1, 7.0 Hz, 1H), 3.68 (s, 3H), 3.86 (s, 3H), 6.76 (s, 1H), 10.58 (s, 1H).^13^C NMR (125 MHz, chloroform-*d*) δ (ppm) 15.5 (CH_3_), 16.6 (CH_3_), 18.6 (CH_2_), 21.7 (CH_2_), 24.8 (CH_3_), 27.5 (CH_2_), 36.5 (CH_2_), 38.3 (CH_2_), 38.4 (C), 43.9 (CH), 47.6 (C), 52.1 (CH_3_), 55.7 (CH_3_), 104.9 (C), 122.1 (C), 127.3 (C), 140.2 (C), 156.6 (C), 161.1 (C), 178.9 (C), 193.0 (CH). IR (film): 2935, 1725, 1681, 1590, 1460, 1404, 1293, 1247, 1135, 1107, 1091, 1041 cm^–1^. HRMS (ESI) *m*/*z*: calcd for C_21_H_39_O_4_ (M+H+) 345.2066, found: 345.2057.

##### (1R,4aS,10aR)-Methyl 7-formyl-6-hydroxy-1,4a,8-trimethyl-1,2,3,4,4a,9,10,10a-octahydrophenanthrene-1-carboxylate (**21**)

To a solution of compound **19** (532 mg, 1.546 mmol) in anhydrous dichloromethane (10 mL) at 0 °C and under argon atmosphere, aluminum bromide (792 mg, 3.0 mmol) was slowly added. The reaction mixture was allowed to gradually warm to room temperature and was stirred at that temperature for 5 h, at which time TLC showed no starting material. Then, the mixture was slowly poured into ice and extracted with ethyl acetate (2 × 30 mL). The combined organic layers were washed with water (3 × 15 mL) and brine (15 mL), dried over anhydrous Na_2_SO_4_, and concentrated under reduced pressure. The crude product was purified by flash chromatography on silica gel (5% AcOEt/hexane) to yield compound **21** (484 mg, 95%) as a white solid; mp 131–133 °C. [α]_D_^25^ = +60.1 (c 1.5, CHCl_3_). ^1^H NMR (400 MHz, chloroform-*d*) δ (ppm) 1.21 (s, 3H), 1.27 (s, 3H), 1.40–1.57 (m, 2H), 1.60–1.87 (m, 5H), 2.17 (dd, *J* = 12.6, 2.1 Hz, 1H), 2.24 (m, 1H), 2.41 (s, 3H), 2.63 (ddd, *J* = 17.0, 11.2, 7.7 Hz, 1H), 2.73 (dd, *J* = 17.0, 7.1 Hz, 1H), 3.68 (s, 3H), 6.77 (s, 1H), 10.37 (s, 1H), 11.82 (s, 1H).^13^C NMR (100 MHz, chloroform-*d*) δ (ppm) 13.5 (CH_3_), 16.7 (CH_3_), 18.6 (CH_2_), 21.6 (CH_2_), 24.6 (CH_3_), 27.3 (CH_2_), 36.6 (CH_2_), 38.1 (CH_2_), 38.4 (C), 43.6 (CH), 47.7 (C), 52.2 (CH_3_), 111.3 (CH), 117.2 (C), 125.5 (C), 140.5 (C), 160.7 (C), 161.1 (C), 179.0 (C), 195.3 (CH). IR (film): 2945, 1725, 1642, 1326, 1248, 1225, 1136, 766 cm^–1^. HRMS (ESI) *m*/*z*: calcd for C_20_H_27_O_4_ (M+H+) 331.1909, found: 331.1906.

##### (1R,4aS,10aR)-Methyl 6-hydroxy-7-(hydroxymethyl)-1,4a,8-trimethyl-1,2,3,4,4a,9,10,10a-octahydrophenanthrene-1-carboxylate (**22**)

Sodium borohydride (60 mg, 1.58 mmol) was added to a stirred solution of **21** (265 mg, 0.80 mmol) in ethanol (6 mL), previously cooled at 0 °C, and the mixture was stirred at room temperature for 1 h. Then, it was cooled at 0 °C and quenched with saturated aqueous NH_4_Cl (2 mL). The solvent was removed under vacuum and AcOEt–H_2_O (20, 10 mL), and the phases were shaken and separated. The aqueous phase was extracted again with AcOEt (2 × 20 mL). The combined organic phases were washed with water (2 × 10 mL), brine (10 mL), dried over anhydrous Na_2_SO_4_, and filtered. Removal of the solvent under vacuum afforded a crude product that was purified by flash chromatography on silica gel (30% AcOEt/hexane) to yield compound **22** as a white solid (220 mg, 83%); mp 98–101 °C. [α]_D_^25^ = +42.3 (c 1.7, CHCl_3_). ^1^H NMR (400 MHz, chloroform-*d*) δ (ppm) 1.20 (s, 3H), 1.26 (s, 3H), 1.32–1.53 (m, 2H), 1.55–1.83 (m, 5H), 2.05 (s, 3H), 2.21 (br d, *J* = 14.5 Hz, 1H), 2.21 (br d, *J* = 12.3Hz, 1H), 2.57 (ddd, *J* = 16.9, 11.1, 7.5 Hz, 1H), 2.68 (dd, *J* = 16.9, 6.9 Hz, 1H), 3.67 (s, 3H), 4.92 (s, 2H), 6.70 (s, 1H), 7.56 (s, 1H). ^13^C NMR (100 MHz, chloroform-*d*) δ (ppm) 14.7 (CH_3_), 16.6 (CH_3_), 18.7 (CH_2_), 22.0 (CH_2_), 25.0 (CH_3_), 28.1 (CH_2_), 36.7 (CH_2_), 37.4 (C), 38.4 (CH_2_), 44.3 (CH), 47.8 (C), 52.2 (CH_3_), 60.8 (CH_2_), 110.1 (CH), 121.0 (C), 125.4 (C), 134.5 (C), 150.7 (C), 154.2 (C), 179.6 (C). IR (film): 3368, 2931, 1725, 1704, 1604, 1421, 1247, 1135, 1035, 986, 770 cm^–1^. TOF MS ES- [M-H] *m*/*z*: 331.1915 (C_20_H_27_O_4_)_._

##### (4R,4aR,11bS)-Methyl 4,7,11b-trimethyl-9-oxo-1,2,3,4,4a,5,6,8,9,11b-decahydrophenanthro[3,2-b]furan-4-carboxylate (**6**) (Taepeenin F)

Pd(PPh_3_)_4_ (72 mg, 0.062 mmol) and P(*o*-tolyl)_3_ (82 mg, 0.27 mmol) were introduced into a tube, and an argon flow was passed inside the tube for 5 min. Then, anhydrous acetic acid (492 mg, 4.82 mmol), formic acid (235 mg, 5.10 mmol), and, last, a solution of **22** (230 mg, 0.69 mmol) in toluene (5 mL) were added. The resulting mixture was sealed and stirred at 100 °C for 16 h, at which time TLC showed the disappearance of compound **22**. The solvent was evaporated under vacuum to afford a crude product, which was purified by flash chromatography on silica gel (20% AcOEt/hexane) to give compound **6** as a white solid (186 mg, 79%). The spectroscopic data of compound **6** agreed with the literature data.^11,16^ mp 193–195 °C. [α]_D_^25^ = +25.0 (c 0.6, CHCl_3_). ^1^H NMR (400 MHz, chloroform-*d*) δ (ppm) 1.22 (s, 3H), 1.28 (s, 3H), 1.39–1.55 (m, 3H), 1.62–187 (m, 5H), 1.83 (m, 1H), 2.10 (s, 3H), 2.21 (br d, *J* = 14.4 Hz, 1H), 2.24 (br d, *J* = 12.7 Hz, 1H), 2.64 (ddd, *J* = 17.3, 11.2, 7.8 Hz, 1H), 2.73 (dd, *J* = 17.3, 6.9 Hz, 1H), 3.60 (s, 2H), 3.68 (s, 3H), 6.90 (s, 1H). ^13^C NMR (100 MHz, chloroform-*d*) δ (ppm) 16.5 (CH_3_), 16.6 (CH_3_), 18.7 (CH_2_), 21.6 (CH_2_), 25.2 (CH_3_), 27.6 (CH_2_), 32.7 (CH_2_), 36.7 (CH_2_), 37.9 (C), 38.6 (CH_2_), 44.4 (CH), 47.7 (C), 52.1 (CH_3_), 104.2 (CH), 119.9 (C), 129.3 (C), 133.0 (C), 150.8 (C), 152.9 (C), 174.8 (C), 179.1 (C). IR (film): 2926, 2853, 1816, 1731, 1249, 1168, 1136, 1012 cm^–1^. HRMS (ESI) *m*/*z*: calcd for C_21_H_27_O_4_ (M+H+) 343.1909, found: 343.1909.

## Biological Assays

Compounds **6**, **16**, **17**, **21**, and **22** were dissolved before use in DMSO (25% in PBS) at a concentration of 5 mg/mL. A stock solution was frozen and stored at 4 °C. Before the experiments, this solution was diluted in cell-culture medium to the adequate concentrations for each experiment. For the antiproliferative assay in all cell lines, we determined the concentrations of compounds required for 20, 50, and 80% of inhibition cell growth, IC_20_, IC_50_, and IC_80_, concentrations, respectively, to analyze the complete range of cytotoxicity and to determine the graduated or acute response to these compounds. For nitrite assay, subcytotoxic concentrations (3/4IC_50_, 1/2IC_50_, and 1/4IC_50_), were used to ensure that the NO inhibitory potential was not due to a possible cytotoxic effect. The concentrations of 3/4IC_50_, 1/2IC_50_, and 1/4IC_50_ were used for the cell-cycle analysis. All experiments were measured and compared with control (untreated cells after 24, 48, or 72 h of treatment.)

### Cell Culture and Viability Assay

Human colorectal adenocarcinoma cell line HT29 (ECACC no. 9172201; ATCC no. HTB-38), human hepatocarcinome cell line HepG2 (ECACC no. 85011430), mouse melanoma cells B16-F10 (ATCC no. CRL-6475), and murine monocyte/macrophage-like RAW 264.7 cell line (ATCC no, TIB-71) were cultured in DMEM (Dulbecco’s modified Eagle’s medium) supplemented with 2 mM glutamine, 10% heat-inactivated FCS (fetal calf serum), 10 000 units/mL of penicillin and 10 mg/mL of streptomycin, (for all cancer cell lines), 50 μg/mL of gentamicin (only for RAW 264.7 cell line), and incubated at 37 °C, in an atmosphere of 5% CO_2_ and 95% humidity. The culture media were changed every 48 h, and the confluent cultures were separated with a trypsin solution (0.25% EDTA). In all experiments, monolayer cells were grown to 80–90% confluence, in sterile cell culture flasks. All cell lines used were purchased from the cell bank of the University of Granada, Spain.

The cytotoxicity assay was performed by the MTT (3-(4,5-dimethylthiazol-2-yl)-2,5-diphenyltetrazolium bromide) method.^21^ To quantify the cytotoxicity of compounds, the fresh and suspended cells were seeded in 96-well plates to a volume of 100 μL at 6.0 × 10^3^ cells/mL for HT29 and RAW 264.7 cell lines, at 5.0 × 10^3^ cells/mL for B16-F10 cells and 15.0 × 10^3^ for Hep G2. Then, cells were incubated to adhere over 24 h at 37 °C with 5% CO_2_. All tested compounds were prepared in fresh growth medium. After 24 h, an additional 100 μL of medium with the corresponding concentrations of products (only medium in the control wells) was added to the corresponding wells, at various final concentrations (0–100 μg/mL). Finally, after 72 h of incubation at 37 °C with 5% CO_2_, the medium with the compounds were removed and 100 μL of MTT solution (0.5 mg/mL) in 50% of PBS 50% of medium was added to each well. After 1.5 h of incubation, formazan was resuspended in 100 μL of DMSO, and each concentration was tested in triplicate. Relative cell viability, with respect to untreated control cells, was measured by absorbance at 570 nm on an ELISA plate reader (Tecan Sunrise MR20-301, TECAN, Austria). Compounds with low IC50 values (**16** and **22**) were selected for several flow cytometric assays, such as apoptosis, cell-cycle, and mitochondrial membrane potential determination.

### NO Inhibitory Activity

#### Determination of Nitric Oxide Release

Nitrites production was analyzed through the Griess reaction. This nitrite concentration was used as an indicator of NO production.^35^ Briefly, RAW 264.7 cells were plated at 6 × 10^3^ cells cells/well in 24-well cell culture plates and supplemented with 10 μg/mL of lipopolysaccharide (LPS). After 24 h of plating, the cells were incubated for 24 h with compounds **(6**, **16**, **17**, **21**, and **22)** at 3/4IC_50_, 1/2IC_50_, and 1/4IC_50_ concentrations. The supernatants were collected at 48 and 72 h, to determine their nitrite concentrations and/or stored at −80 °C for further use.

The Griess reaction was performed taking 150 μL of supernatant test samples or sodium nitrite standard (0–120 μM) and mixed with 25 μL of Griess reagent A (0.1% N,N-(1-naphthyl) ethylenediaminedihydrochloride) and 25 μL of Griess reagent B (1% sulfanilamide in 5% of phosphoric acid), in a 96-well plate. After 15 min of incubation at room temperature, the absorbance was measured at 540 nm in an ELISA plate reader (Tecan Sunrise MR20–301, TECAN, Austria). The absorbance was referred to a nitrite standard curve to determine the concentration of nitrite in the supernatant of each experimental sample. The percentage of NO production was determined, assigning 100% at the increase between negative control (untreated cells) and positive control (cells only treated with 10 μg/mL of LPS).

#### Cell Cycle

Cellular subpopulations, with different DNA contents, were visualized, using a fluorescence-activated cell sorter (FACS) at 488 nm in an Epics XL flow cytometer (Coulter Corporation, Hialeah, FL, USA). Changes in DNA levels are characteristic of cell-cycle arrest and cell differentiation.^36^ For this assay, 12 × 10^4^ RAW 264.7 murine macrophage/monocyte cells stimulated with LPS were plated in 24-well plates with 1.5 mL of medium and incubated with the compounds under study for 24 h, at 3/4IC_50_ and 1/2IC_50_ concentrations. Several controls were analyzed in parallel. A positive control was performed using cells treated only with LPS stimulation, and a negative control was performed where RAW 264.7 cells were exposed to the tested compounds without lipopolysaccharide stimulation. The treated RAW 264.7 cells (with LPS and tested compounds) were washed twice with PBS and harvested by trypsinization and then were resuspended in TBS 1× (10 mM Tris, 150 mM NaCl), to which Vindelov Buffer (100 mM Tris, 100 MmNaCl, 10 mg/mL Rnasa, 1 mg/mL PI, pH 8) was subsequently added. The samples were allowed to stand for 15 min on ice. Immediately before FACS analysis, cells were stained with 20 μL of 1 mg/mL PI solution. Data were analyzed to determine the percentage of cells in each cell-cycle phase (G0/G1, S, and G2/M).

### Antitumor Activity

#### B16-F10 Cell-Cycle Analysis

The method used to quantify the amount of DNA in the different phases of the cell cycle (G0/G1, S, and G2/M) was performed by flow cytometry after propidium iodide staining (PI).^37^ For this assay, B16-F10 cells were plated and after 24 h were treated or not (control) with IC_50_ and IC_80_ concentrations of the different compounds selected for 48 h. After treatment, the cells were washed twice with PBS, trypsinized, and resuspended in 1× TBS (10 mM Tris and 150 mM NaCl). After that, Vindelov buffer (100 mM Tris, 100 mM NaCl, 10 mg/mL Rnase, and 1 mg/mL PI, at pH 8) was added. Cells were stored on ice and, just before measurement, were stained with 20 μL of 1 mg/mL PI solution. Approximately, 10 × 10^3^ cells were analyzed in each experiment. The experiments were performed three times with two replicates per assay. Finally, samples were analyzed using a flow cytometer, and the number of cells in each stage of the cell cycle was estimated by fluorescence-associated cell sorting (FACS) at 488 nm in an Epics XL flow cytometer (Coulter Corporation, Hialeah, FL, USA).

#### Annexin V-FITC/Propidium Iodide Flow Cytometry Analysis

To confirm the proapoptotic effect of compounds **16** and **22**, annexin V and PI double staining was detected with flow cytometry. An early indicator of apoptosis is the translocation of the membrane phospholipid phosphatidylserine from the cytoplasmic interface to the external surface of the plasmatic membrane. The phospholipid phosphatidylserine that accumulates on the external plasmatic membrane can be detected by annexin V/PI is a fluorescent dye that binds to the nuclei of dead cells. Apoptosis was assessed by flow cytometry using a FACScan (fluorescence-activated cell sorter) flow cytometer (Coulter Corporation, Hialeah, FL, USA).^37,38^ For this assay, 5 × 10^4^ B16-F10 cells were plated in 24-well plates with 1.5 mL of medium and incubated for 24 h. Subsequently, the cells were treated with the selected compounds in triplicate for 24, 48, and 72 h at their corresponding IC_50_ concentration. Cells were collected and resuspended in a binding buffer (10 mM HEPES/NaOH, pH 7.4, 140 mM NaCl, 2.5 mM CaCl_2_). Annexin V-FITC conjugate (1 μg/mL) was then added and incubated for 15 min at room temperature in darkness. Just before the analysis by flow cytometry, cells were stained with 5 μL of 1 mg/mL PI solution. In each experiment, approximately 10 × 10^3^ cells were analyzed, and the experiment was duplicated twice.

#### Flow Cytometry Analysis of the Mitochondrial Membrane Potential

The electrochemical gradient across the mitochondrial membrane was investigated by analytical flow cytometry, using dihydrorhodamine (DHR). DHR is oxidized in contact with living cells, forming a highly fluorescent product called rhodamine (Rh 123). The emitted fluorescence can be monitored by fluorescence spectroscopy using excitation and emission wavelengths of 500 and 536 nm, respectively.^37−39^ 5 × 10^4^ B16-F10 cells were plated in 24-well plates, incubated for 24 h, and treated with the selected compounds for 48 h at their corresponding IC_50_ concentrations. After treatment, the culture medium was renewed by adding fresh medium with DHR to a final concentration of 5 mg/mL. Cells were incubated for 1 h at 37 °C in an atmosphere of 5% CO_2_ and 95% humidity and subsequently washed and resuspended in PBS with 5 μg/mL of PI. The fluorescence intensity was measured using a FACScan flow cytometer (fluorescence-activated cell sorter). The experiments were performed three times with two replicates per assay.

#### Statistical Analysis

Cytotoxicity experimental data were fitted to a sigmoidal function (*y* = ymax/(*x*/*a*)^−*b*^) by nonlinear regression. IC_20_, IC_50_, and IC_80_ values (concentrations that cause 20%, 50%, and 80% of cell viability inhibition respectively) were obtained by interpolation. These analyses were performed using SigmaPlot 12.5 software. Similar analyses were performed to obtain the IC_50_ of NO production. All data shown here are representative of at least two independent experiments performed in triplicate. All quantitative data were summarized as the mean ± standard deviation (SD).

## Supporting Information Available

Figures S1–S3 and ^1^H and ^13^C NMR spectra of compounds (PDF)
