# Supplementary material for: Semisynthesis and Evaluation of Anti-Inflammatory Activity of the Cassane-Type Diterpenoid Taepeenin F and of Some Synthetic Intermediates
Source: J Nat Prod. 2022 Oct 10;85(10):2372–84. doi: 10.1021/acs.jnatprod.2c00578 (PMC9623576; doi:10.1021/acs.jnatprod.2c00578)
Supplement: Supplementary file 1 — np2c00578_si_001.pdf [file np2c00578_si_001.pdf]

SUPPORTING INFORMATION

Semisynthesis and evaluation of anti-inflammatory activity of the cassane-type diterpenoid taepeenin F and of some synthetic intermediates.

Houda Zentar[†]; Fatin Jannus[¥]; Pilar Gutierrez[†]; Marta Medina-O'Donnell[¥]; José Antonio Lupiáñez[¥]; Fernando J. Reyes-Zurita[¥]*; Enrique Alvarez-Manzaneda[†]; Rachid Chahboun[†]*

[†]Departamento de Química Orgánica, Facultad de Ciencias, Instituto de Biotecnología,

Universidad de Granada, 18071 Granada, Spain

[¥]Departamento de Bioquímica y Biología Molecular I, Facultad de Ciencias, Universidad de Granada, 18071, Granada, Spain

Table of contents

Figure S1. Citotoxicity on RAW 264.7 cell line……………………………..…….S2

Figure S2. RAW 264.7 cell cycle arrest and distribution…..........………………S3

Figure S3. Citotoxicity on cancer cell lines …..................……………………….S4

^{1}H and ^{13}C NMR spectra of compounds ……………..…………………………S5-S24

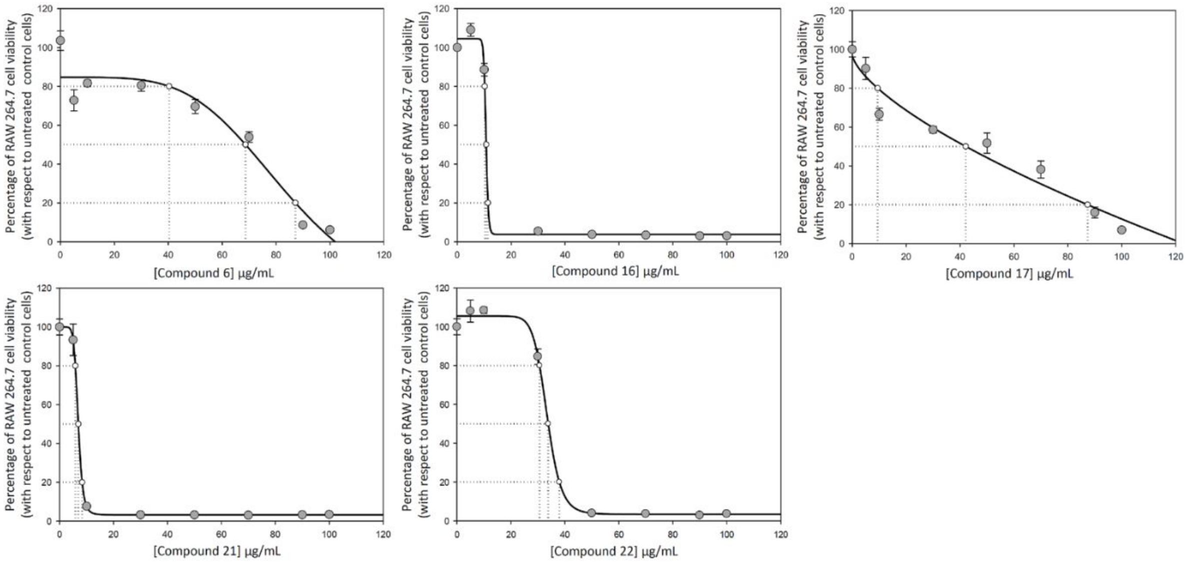

Figure S1. Effect of compounds **6**, **16**, **17**, **21** and **22**, on cell proliferation of RAW 264.7 macrophage murine cells. After treatment with the compounds in the range of concentration from 0 to 100 µg/mL, each point represents the mean value ± S.D. of at least two independent experiments performed in triplicate. IC_{20}, IC_{50}, and IC_{80} are the concentrations required for growth inhibition of 20%, 50% and 80%.

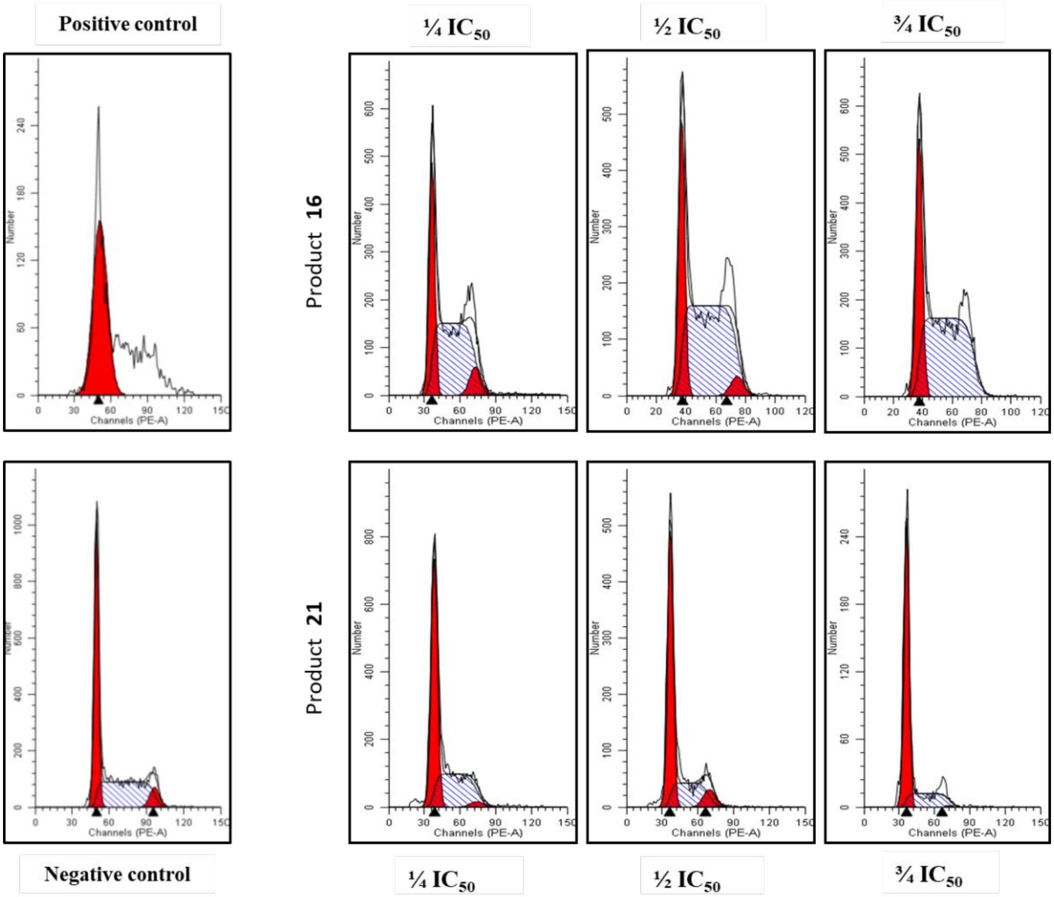

Figure S2. Cell-cycle histograms of RAW 264.7 macrophage murine cells that were treated with LPS for 72 h, to induced inflammatory process. Then treated with compounds **16** and **21**, at their ¼ $\times IC_{50}$, ½ $\times$ IC_{50} and ¾ $\times$ IC_{50} concentrations. In addition, results obtained for negative control (untreated cells) and positive control (cells only treated with LPS) are showed. Data represent the mean ± S.D. of at least two independent experiments performed in triplicate.

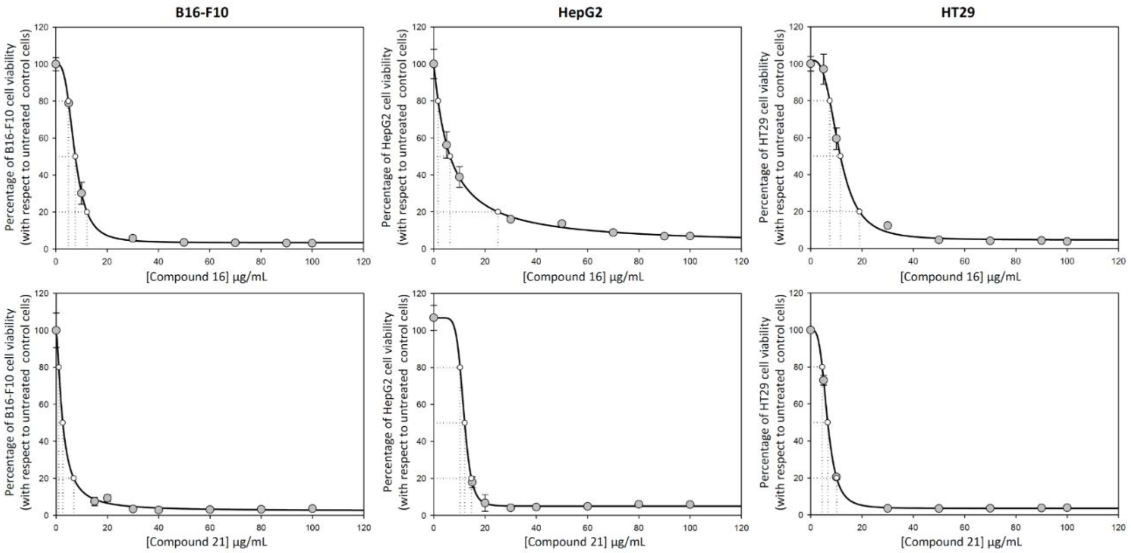

Figure S3. Effects of compounds **16** and **21**, on the viability of B16-F10, HT29, and HepG2 cancer cells, after treatment with the compounds for 72 h in a range of 0 to 100 µg/mL, each point represents the mean value ± SD of at least two independent experiments performed in triplicate

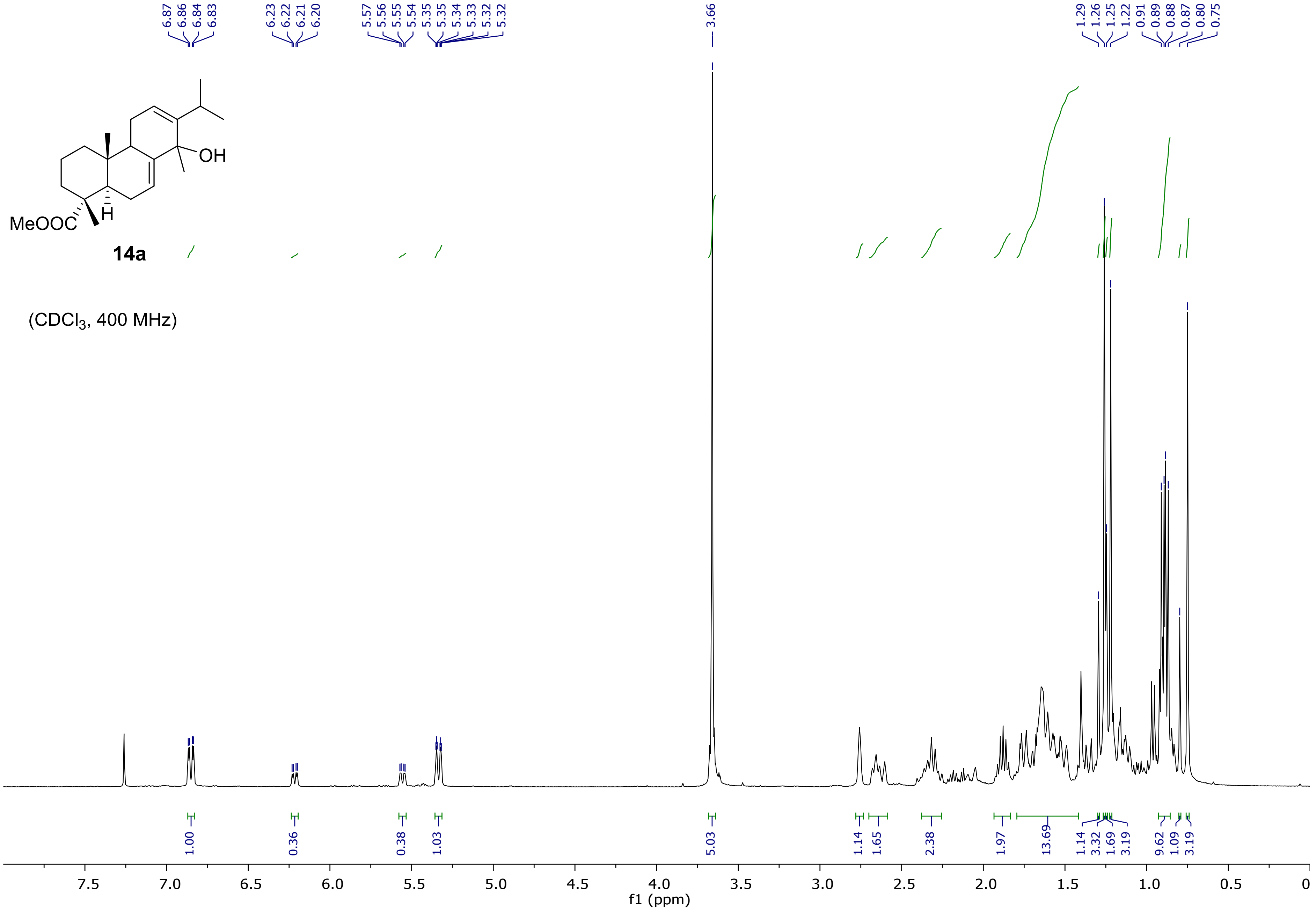
6.87 6.86 6.84 6.83
6.23 6.22 6.21 6.20
5.57 5.56 5.55 5.54 5.35 5.35 5.34 5.33 5.32 5.32
3.66
1.29 1.26 1.25 1.22 0.91 0.89 0.88 0.87 0.80 0.75
OH
MeOOC
H
14a
(CDCl$_3$, 400 MHz)
1.00
0.36
0.38
1.03
5.03
1.14
1.65
2.38
1.97
13.69
1.14
3.32
1.69
3.19
9.62
1.09
3.19
7.5 7.0 6.5 6.0 5.5 5.0 4.5 4.0 3.5 3.0 2.5 2.0 1.5 1.0 0.5 0
f1 (ppm)

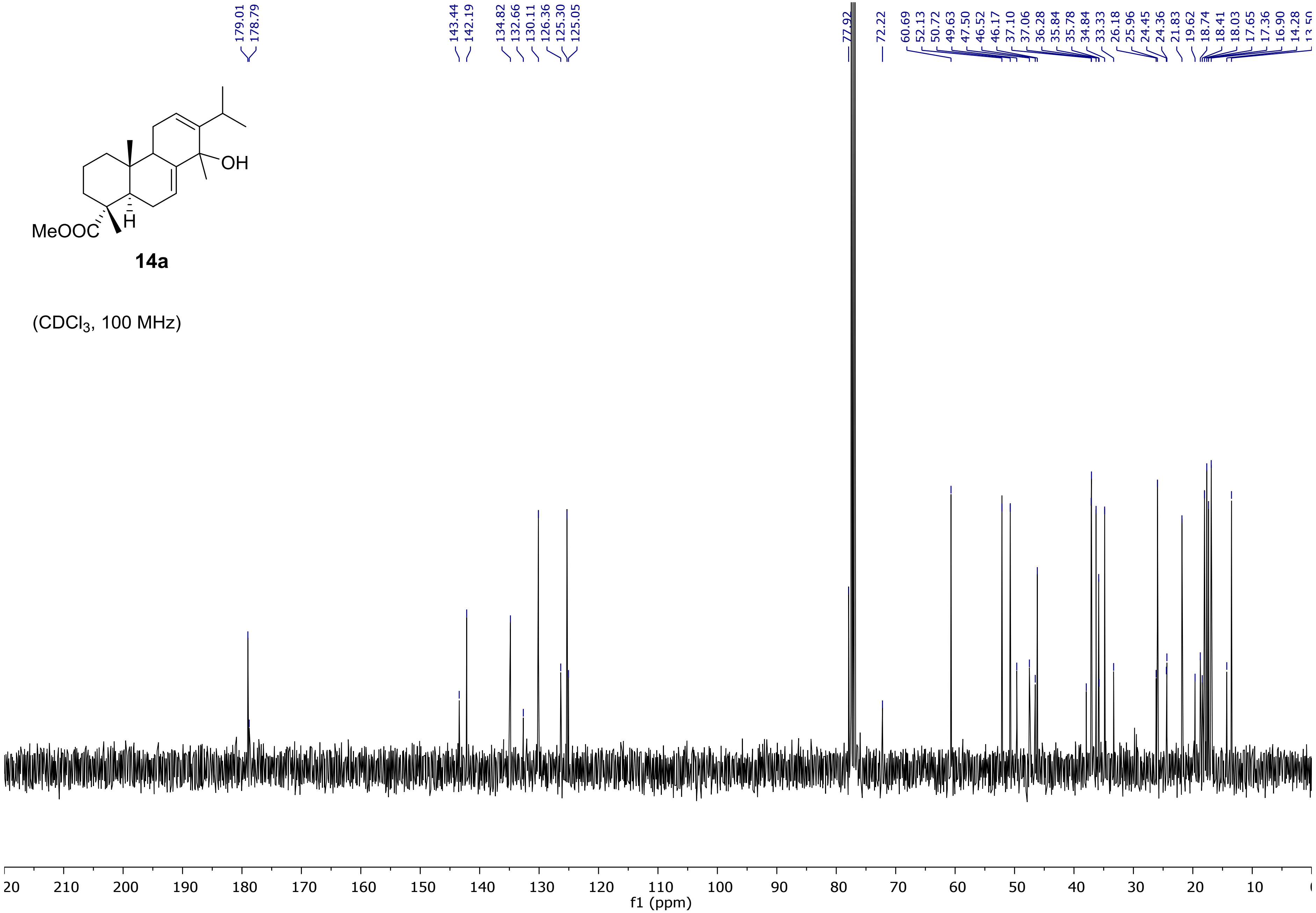

14a

(CDCl$_3$, 100 MHz)

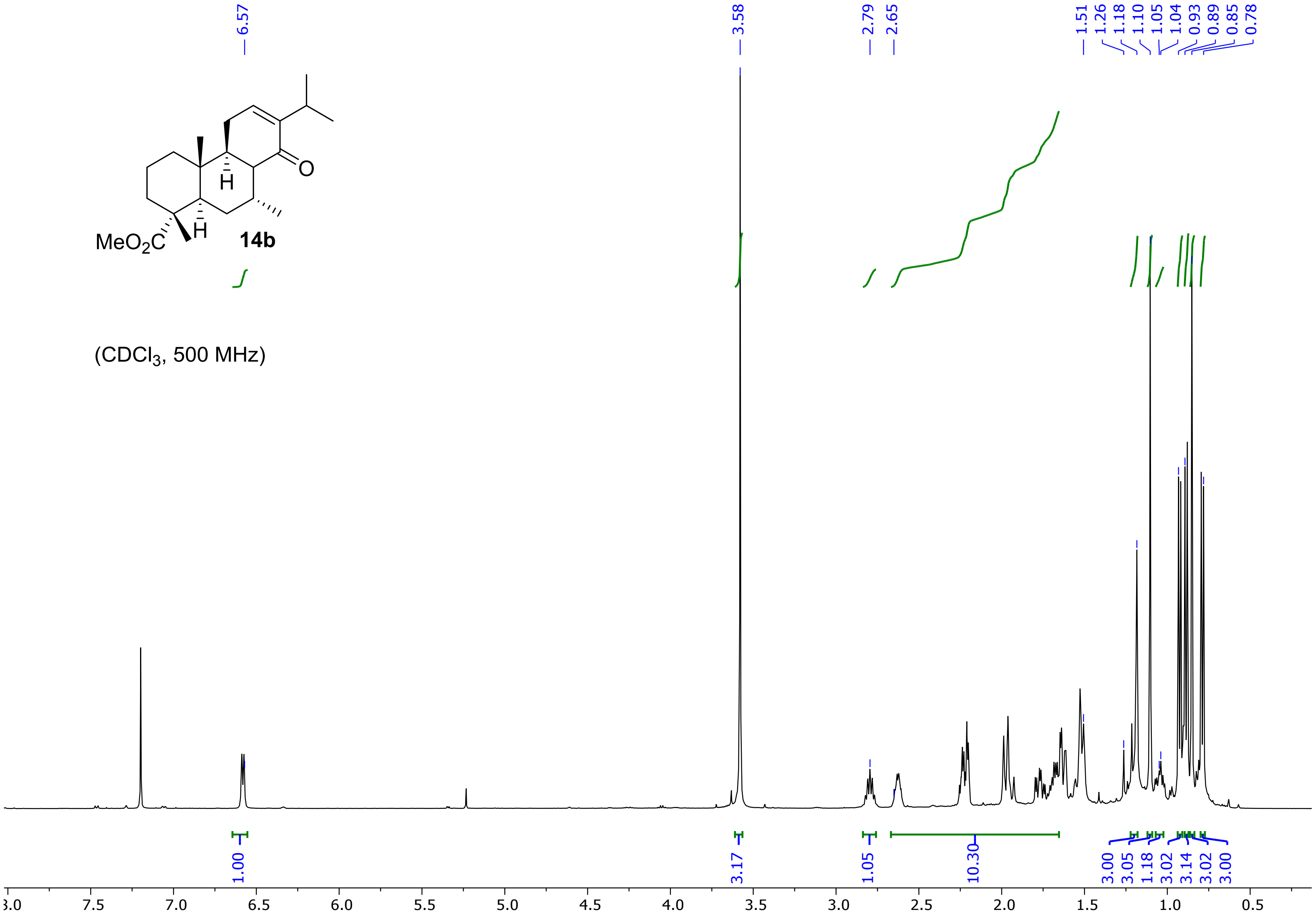

6.57
3.58
2.79
2.65
1.51
1.26
1.18
1.10
1.05
1.04
0.93
0.89
0.85
0.78
MeO2C
14b
(CDCl3, 500 MHz)
1.00
3.17
1.05
10.30
3.00
3.05
1.18
3.02
3.14
3.02
3.00
8.0
7.5
7.0
6.5
6.0
5.5
5.0
4.5
4.0
3.5
3.0
2.5
2.0
1.5
1.0
0.5

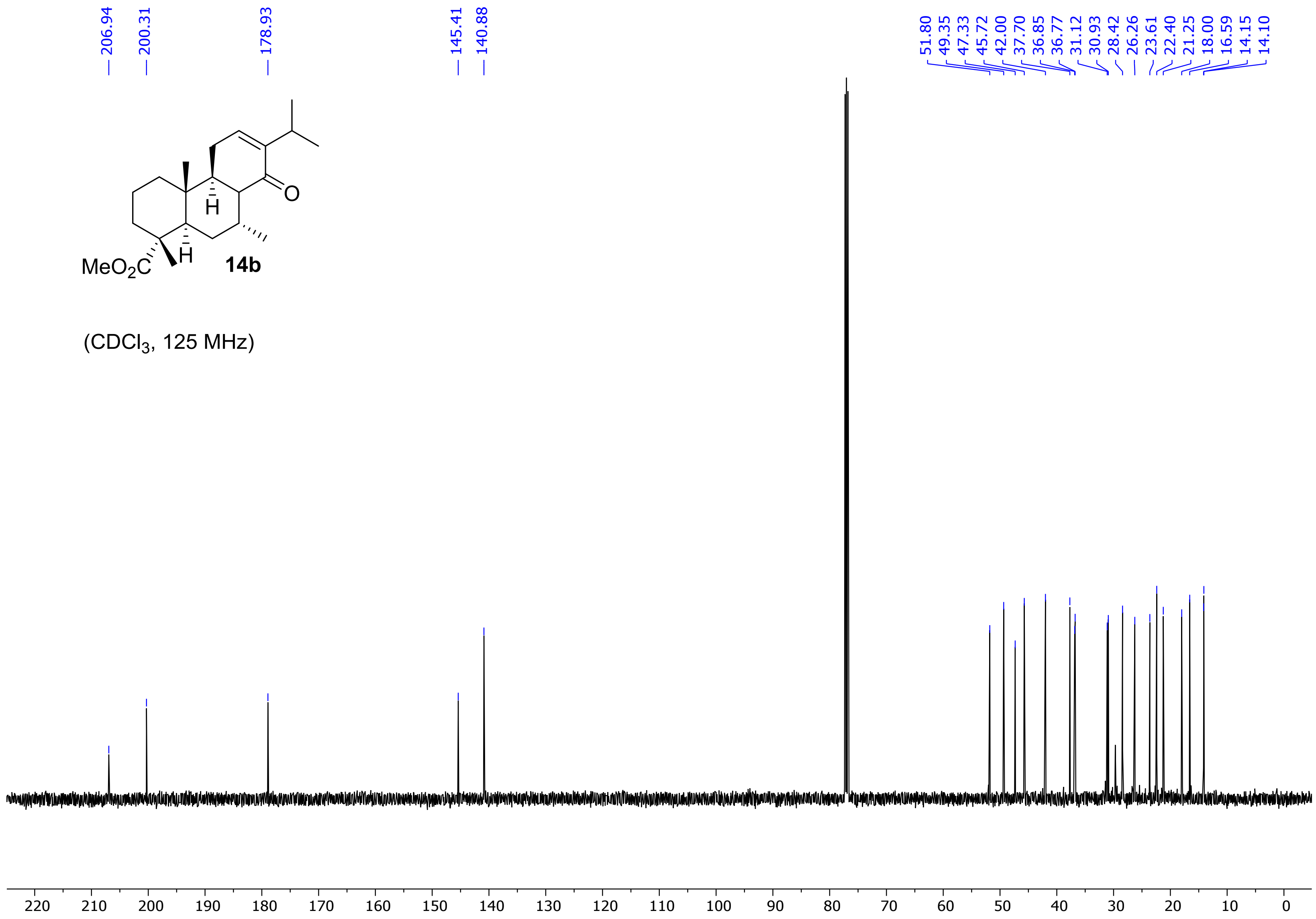
206.94
200.31
178.93
145.41
140.88
51.80
49.35
47.33
45.72
42.00
37.70
36.85
36.77
31.12
30.93
28.42
26.26
23.61
22.40
21.25
18.00
16.59
14.15
14.10
MeO2C
H
H
O
14b
(CDCl3, 125 MHz)
220
210
200
190
180
170
160
150
140
130
120
110
100
90
80
70
60
50
40
30
20
10
0

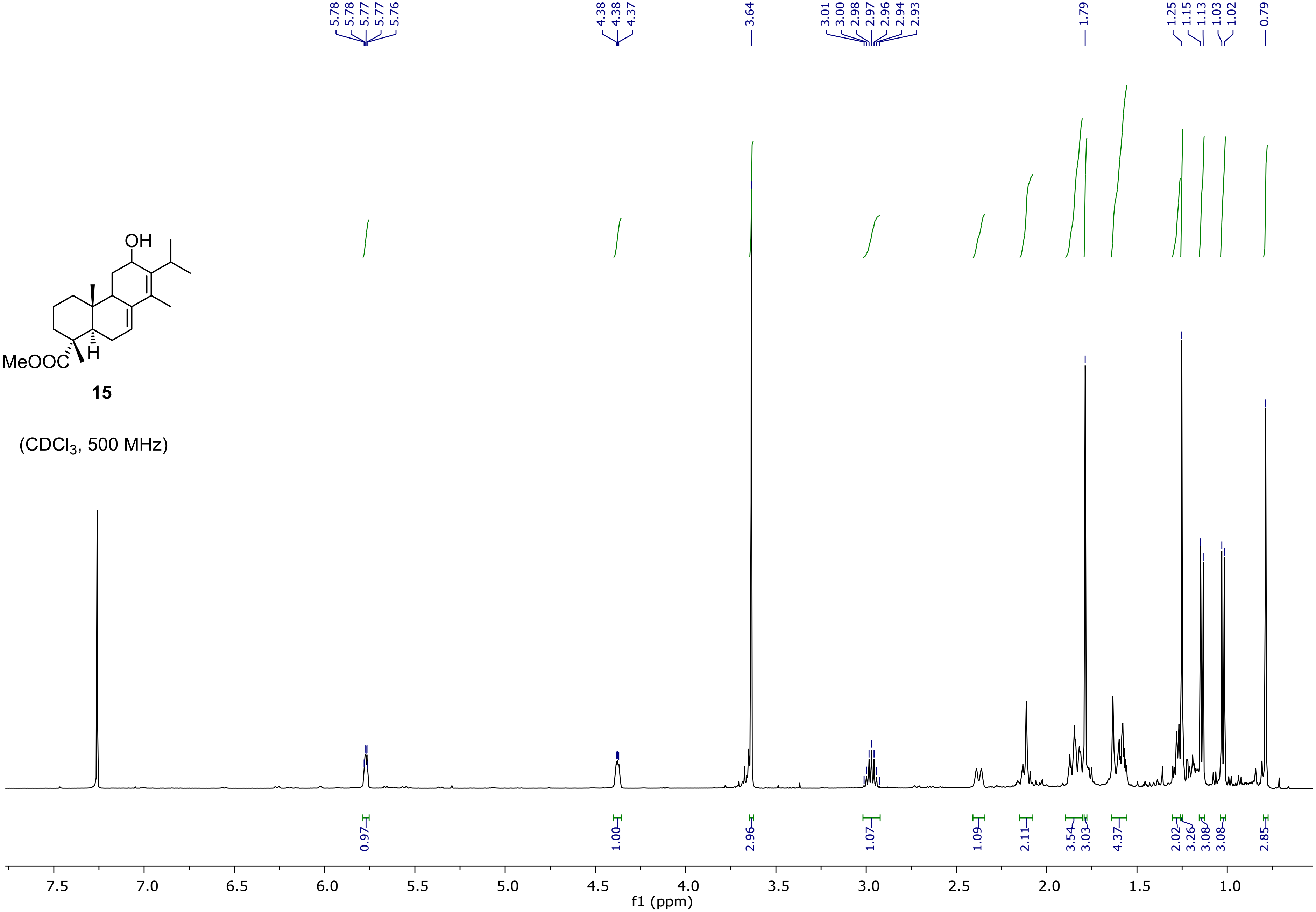

15

($CDCl_3$, 500 MHz)

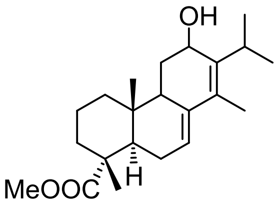

15

($CDCl_3$, 125 MHz)

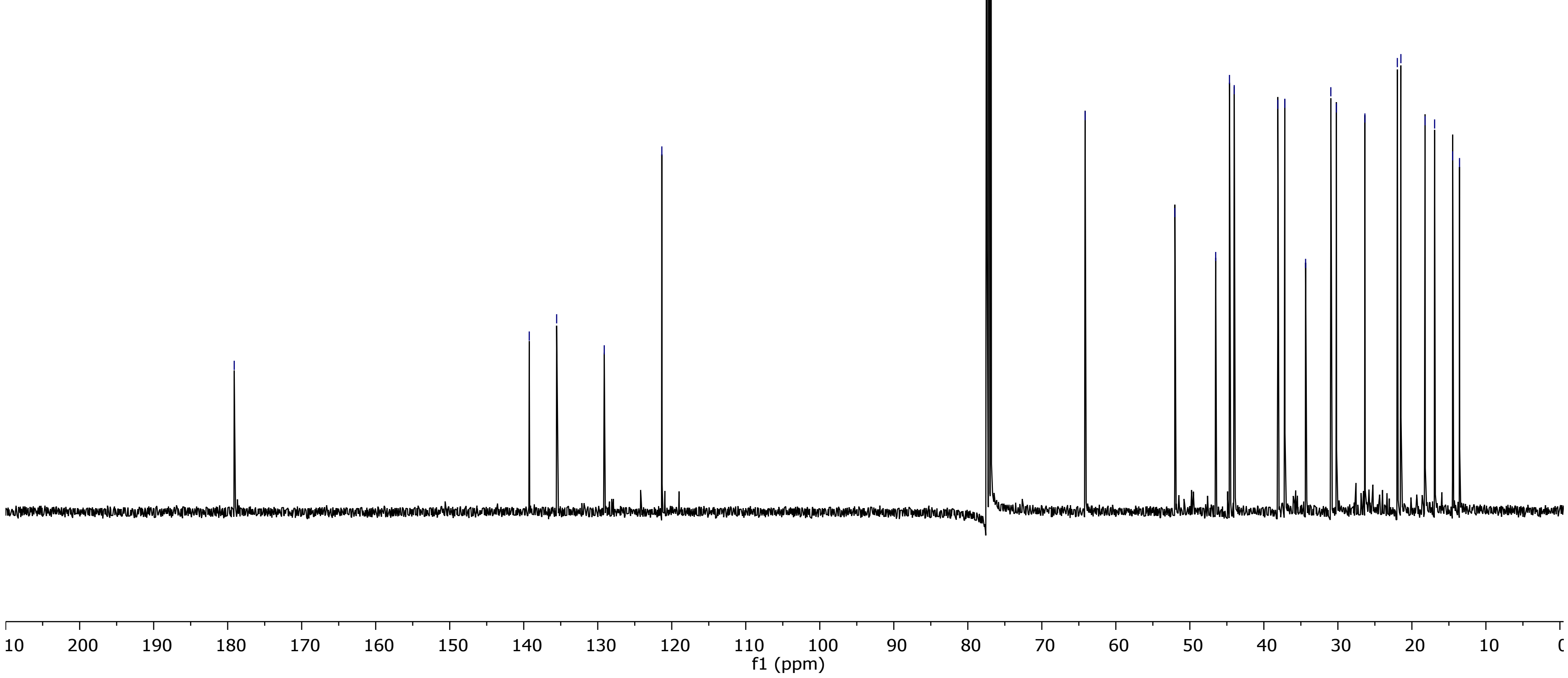

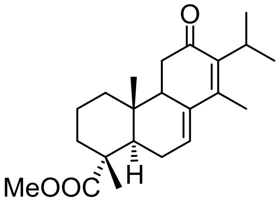

16

($CDCl_3$, 500 MHz)

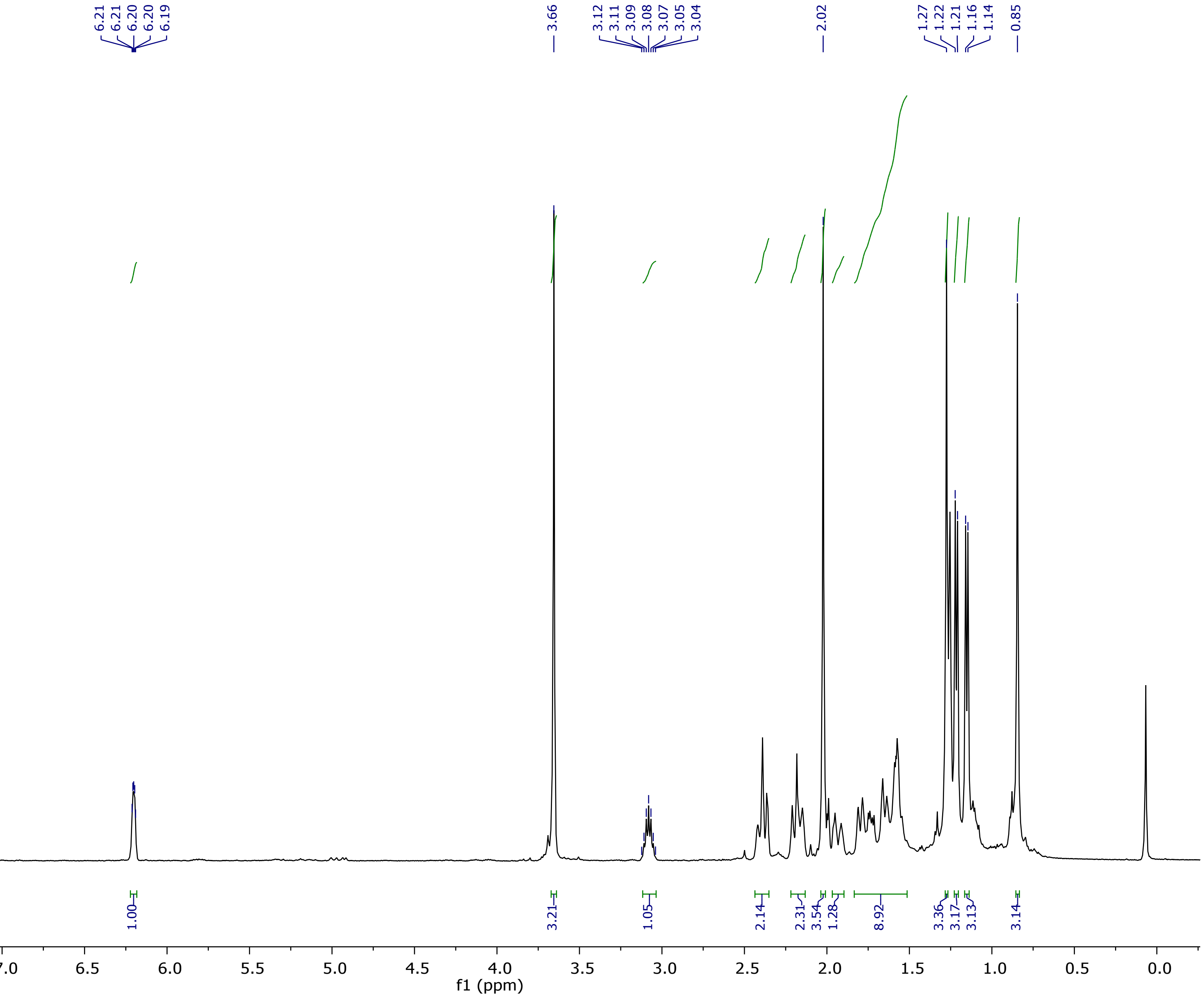

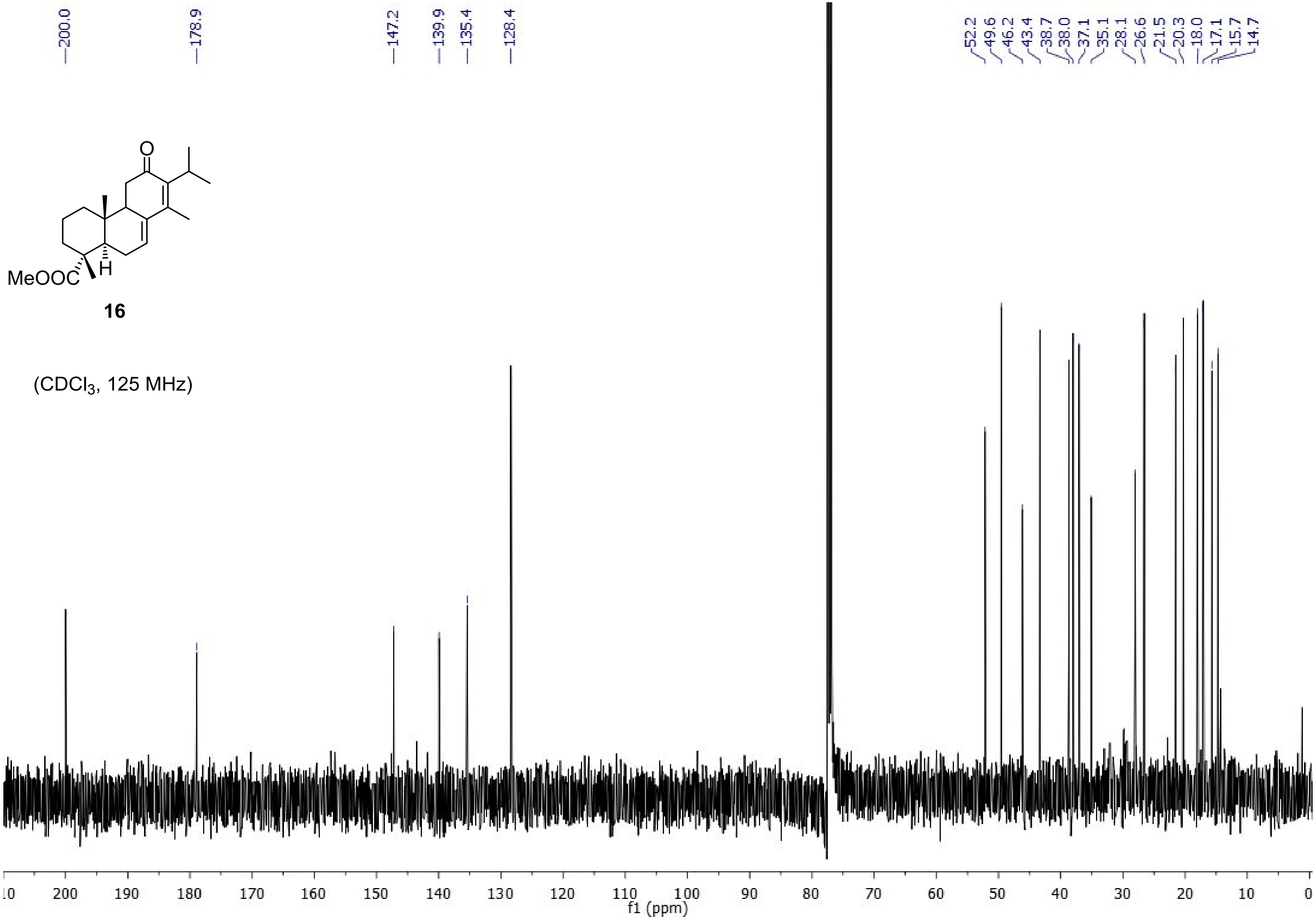

16

($CDCl_3$, 125 MHz)

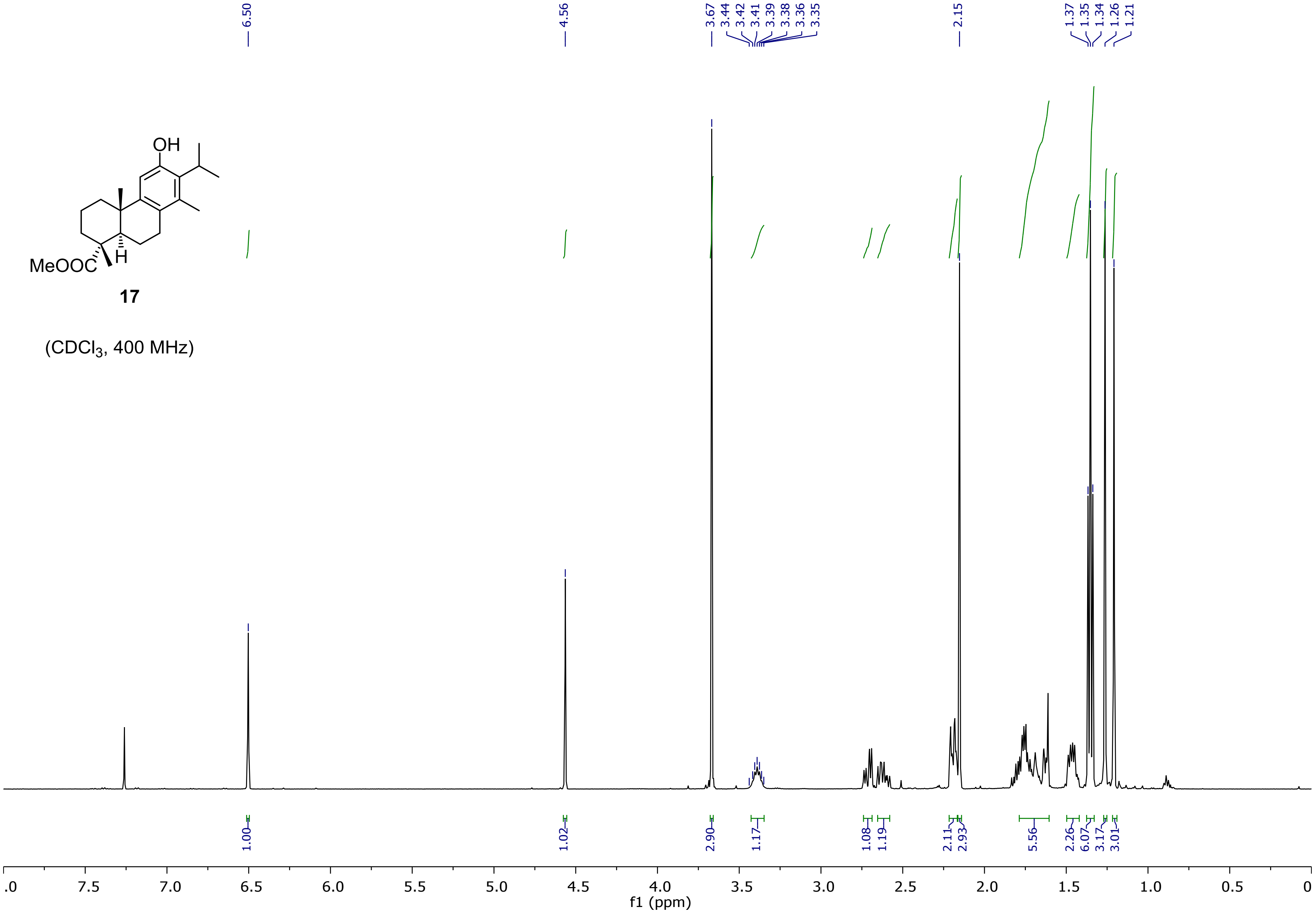
6.50
4.56
3.67 3.44 3.42 3.41 3.39 3.38 3.36 3.35
2.15
1.37 1.35 1.34 1.26 1.21
OH
MeOOC
H
17
(CDCl3, 400 MHz)
1.00
1.02
2.90
1.17
1.08
1.19
2.11
2.93
5.56
2.26
6.07
3.17
3.01
.0 7.5 7.0 6.5 6.0 5.5 5.0 4.5 4.0 3.5 3.0 2.5 2.0 1.5 1.0 0.5 0
f1 (ppm)

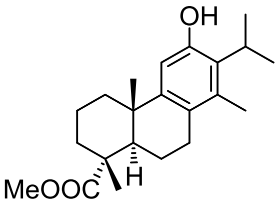

17

($CDCl_3$, 100 MHz)

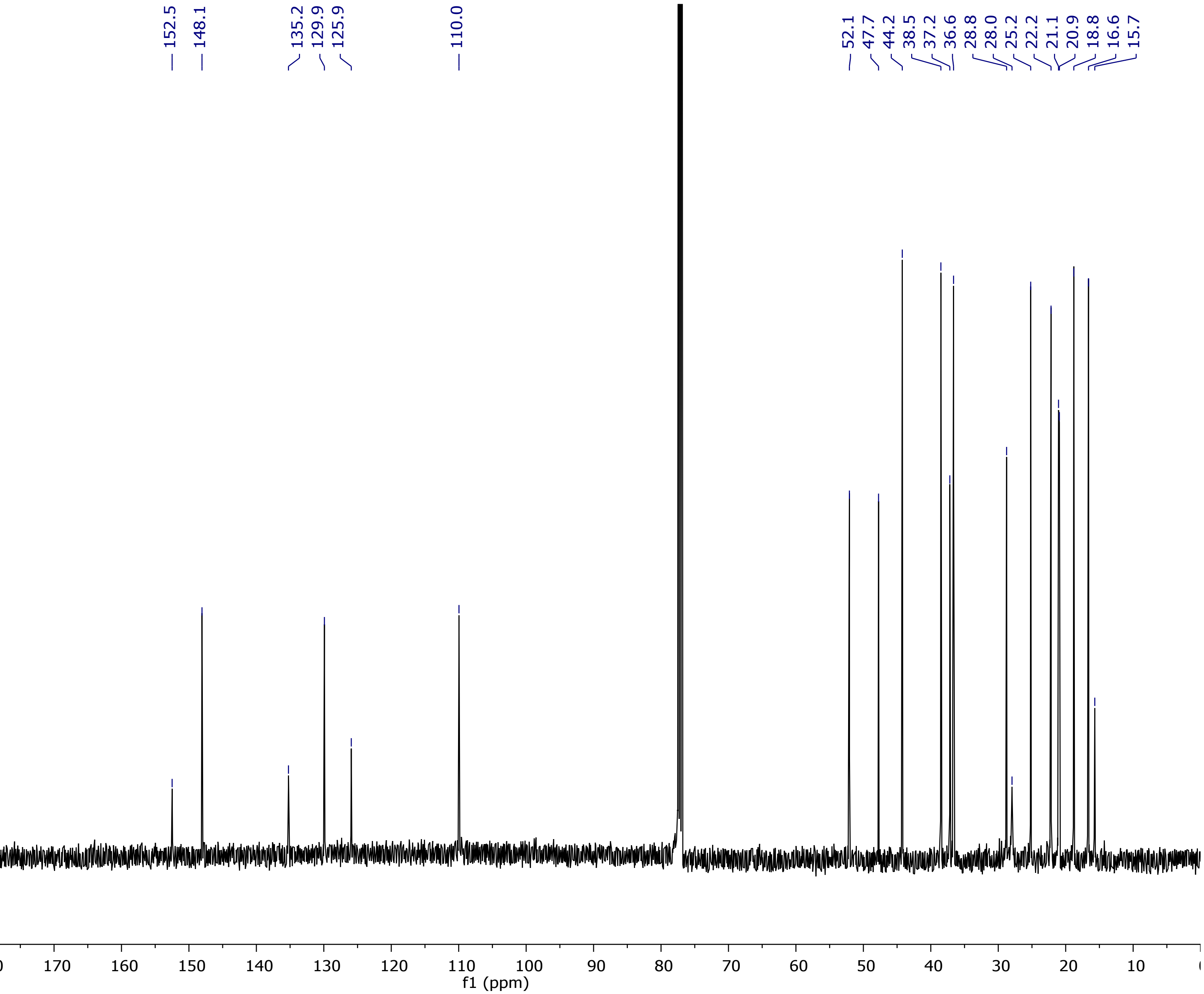

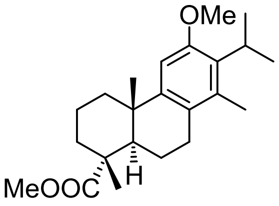

18

(CDCl$_3$, 400 MHz)

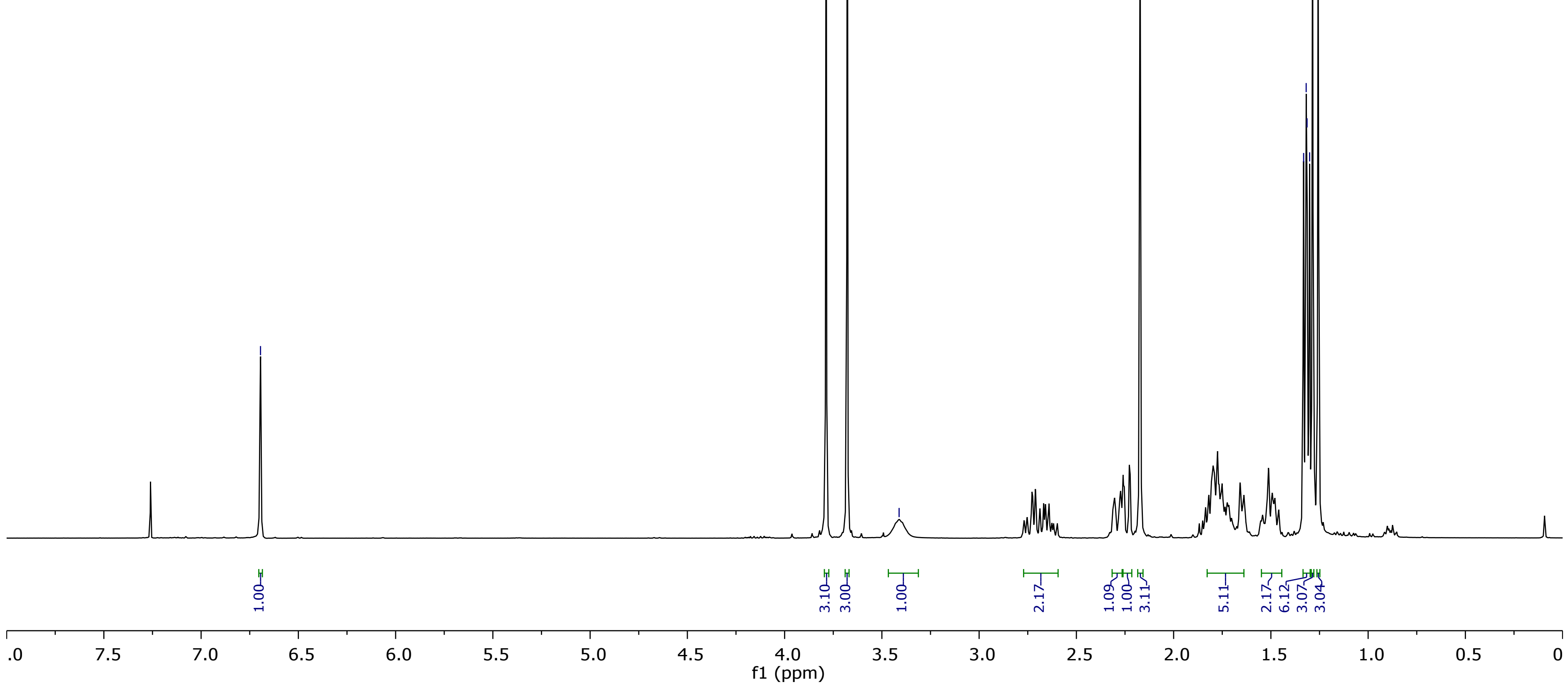

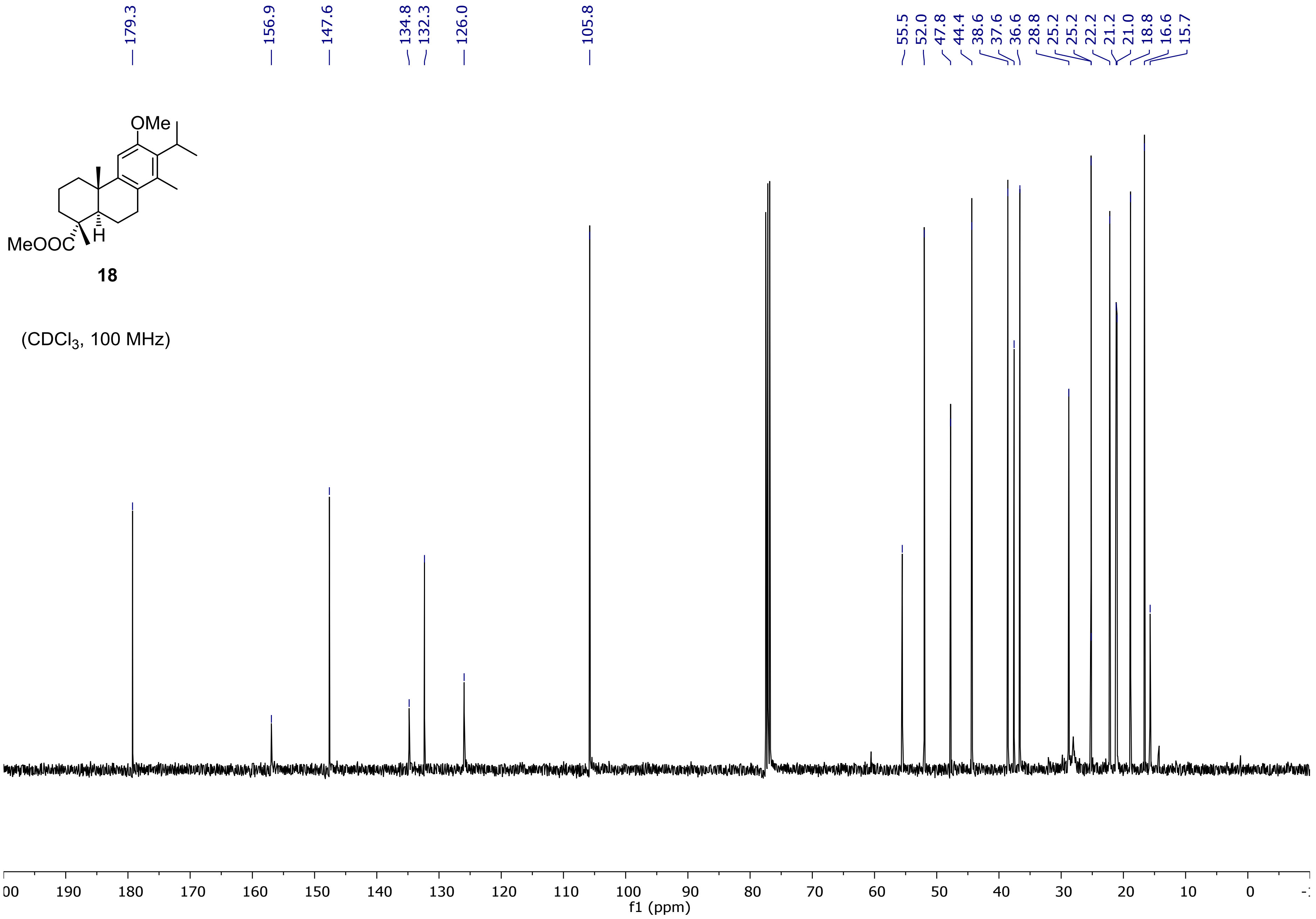
179.3
156.9
147.6
134.8
132.3
126.0
105.8
55.5
52.0
47.8
44.4
38.6
37.6
36.6
28.8
25.2
25.2
22.2
21.2
21.0
18.8
16.6
15.7
OMe
MeOOC
H
(CDCl$_3$, 100 MHz)
f1 (ppm)

18

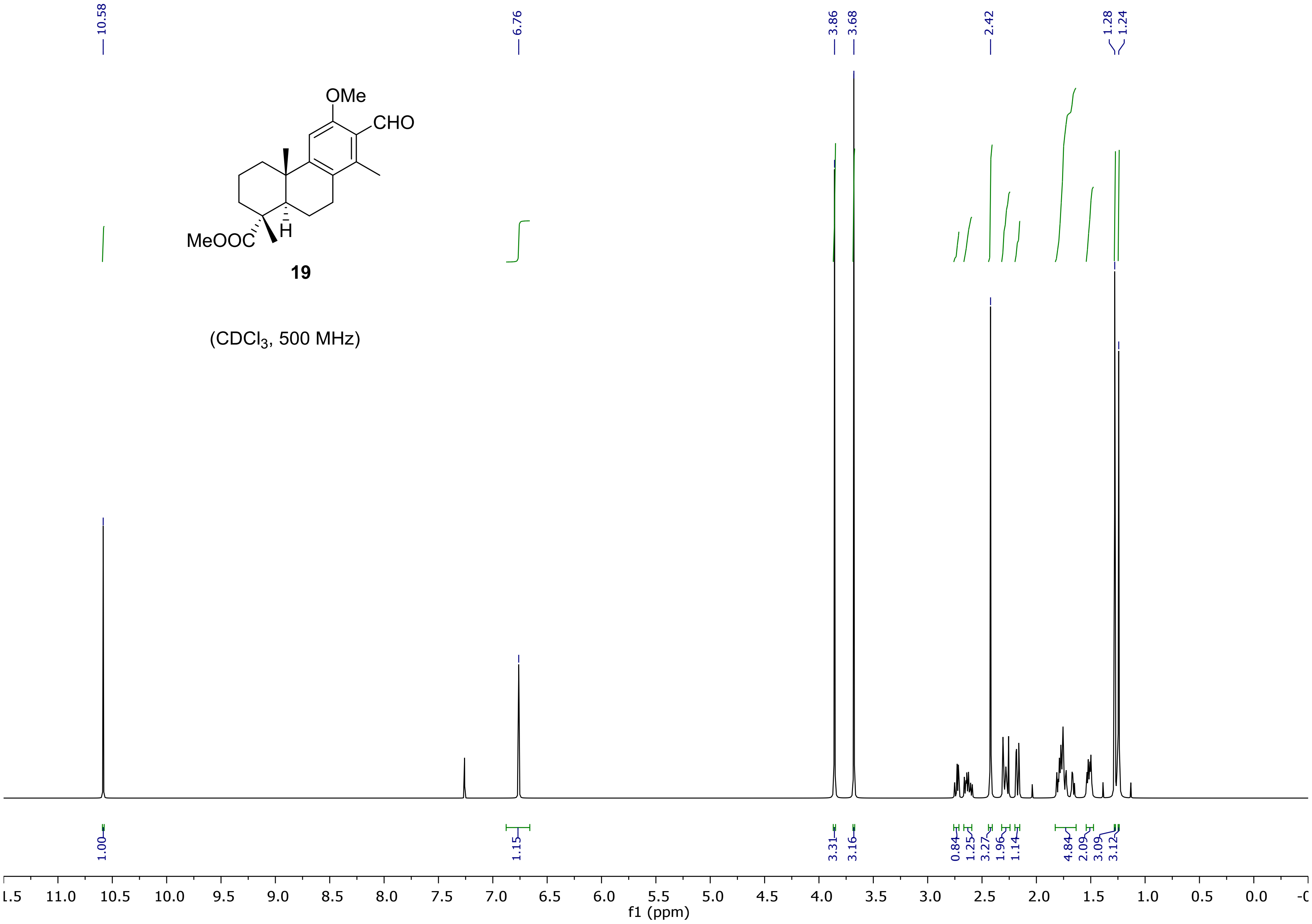
10.58
6.76
3.86
3.68
2.42
1.28
1.24
OMe
CHO
MeOOC
H
19
(CDCl$_3$, 500 MHz)
1.00
1.15
3.31
3.16
0.84
1.25
3.27
1.96
1.14
4.84
2.09
3.09
3.12
11.0 10.5 10.0 9.5 9.0 8.5 8.0 7.5 7.0 6.5 6.0 5.5 5.0 4.5 4.0 3.5 3.0 2.5 2.0 1.5 1.0 0.5 0.0
f1 (ppm)

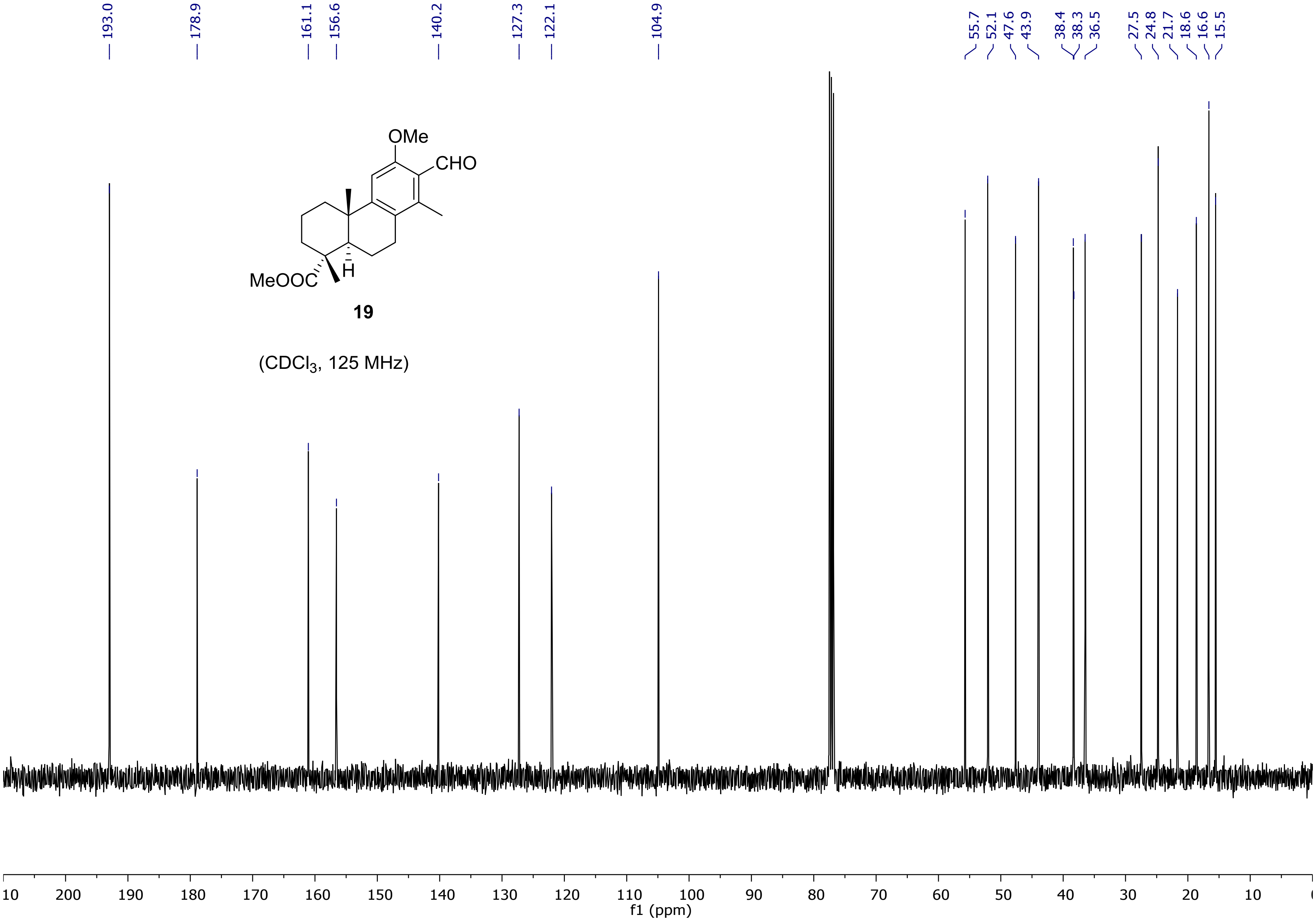

193.0
178.9
161.1
156.6
140.2
127.3
122.1
104.9
55.7
52.1
47.6
43.9
38.4
38.3
36.5
27.5
24.8
21.7
18.6
16.6
15.5
OMe
CHO
MeOOC
H
19
(CDCl3, 125 MHz)
10 200 190 180 170 160 150 140 130 120 110 100 90 80 70 60 50 40 30 20 10
f1 (ppm)

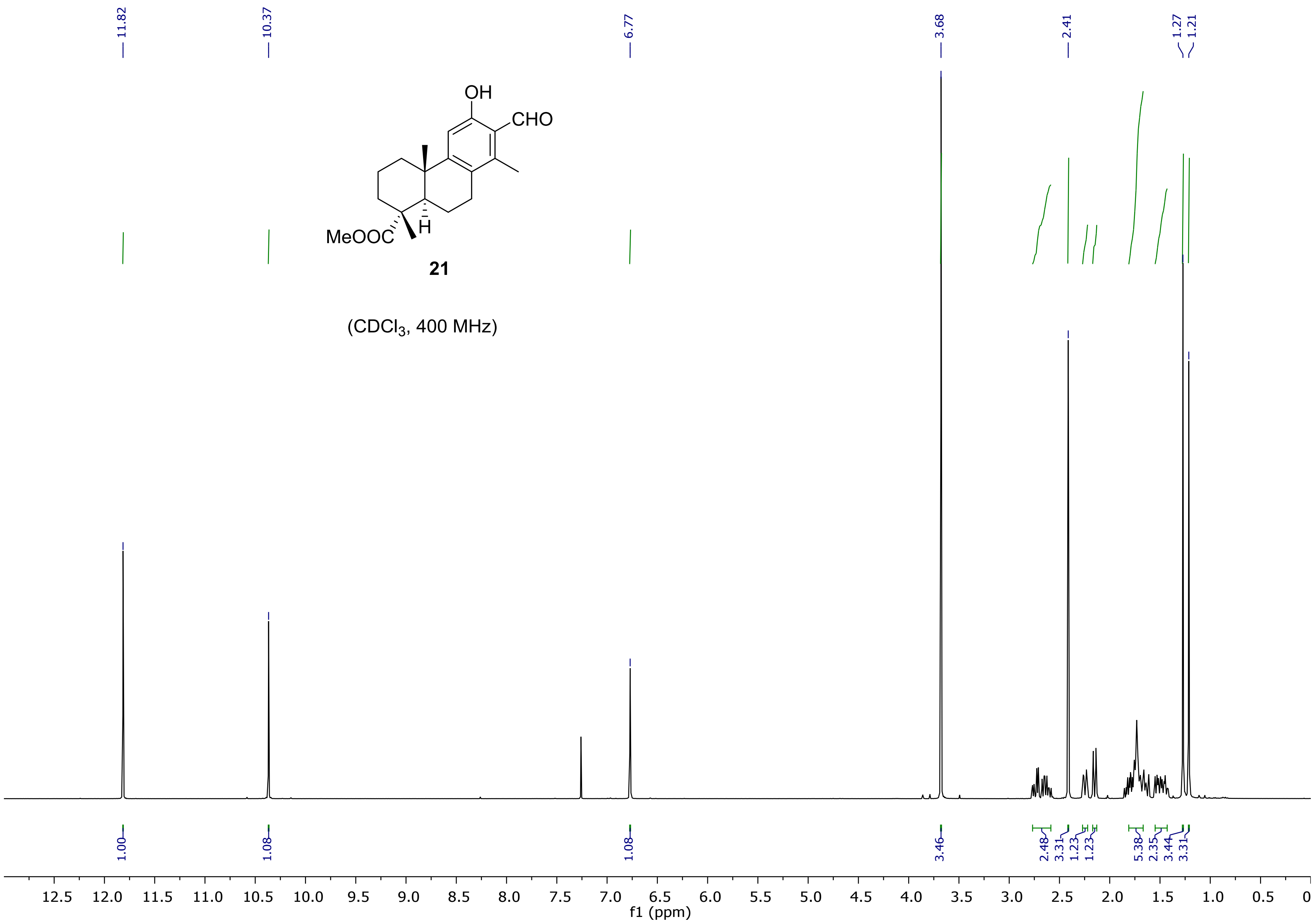

11.82
10.37
6.77
3.68
2.41
1.27
1.21
OH
CHO
MeOOC
H
21
(CDCl$_3$, 400 MHz)
1.00
1.08
1.08
3.46
2.48
3.31
1.23
1.23
5.38
2.35
3.44
3.31
12.5 12.0 11.5 11.0 10.5 10.0 9.5 9.0 8.5 8.0 7.5 7.0 6.5 6.0 5.5 5.0 4.5 4.0 3.5 3.0 2.5 2.0 1.5 1.0 0.5 0
f1 (ppm)

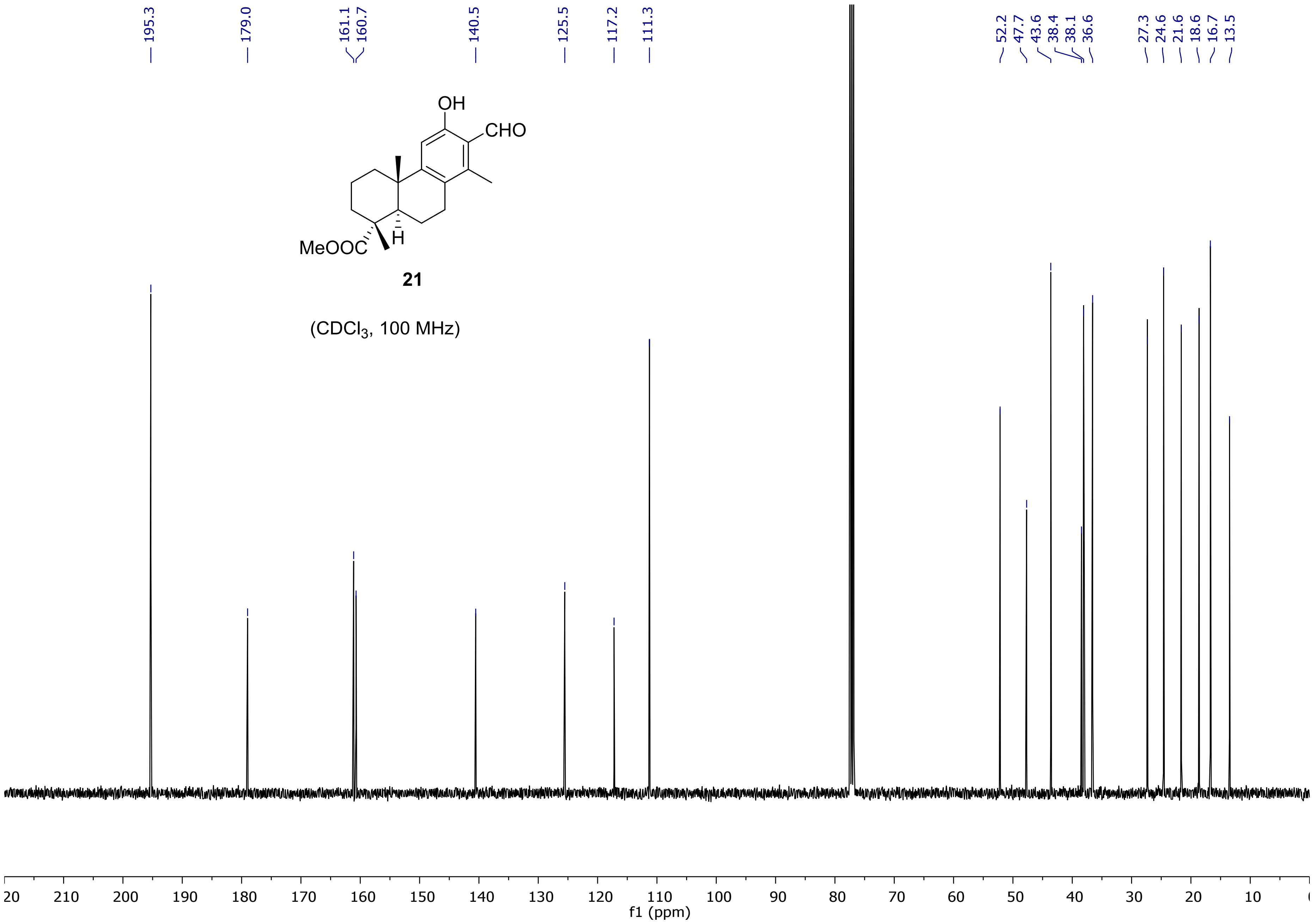
195.3
179.0
161.1
160.7
140.5
125.5
117.2
111.3
52.2
47.7
43.6
38.4
38.1
36.6
27.3
24.6
21.6
18.6
16.7
13.5
OH
CHO
MeOOC
H
21
(CDCl3, 100 MHz)
20
210
200
190
180
170
160
150
140
130
120
110
100
90
80
70
60
50
40
30
20
10
f1 (ppm)

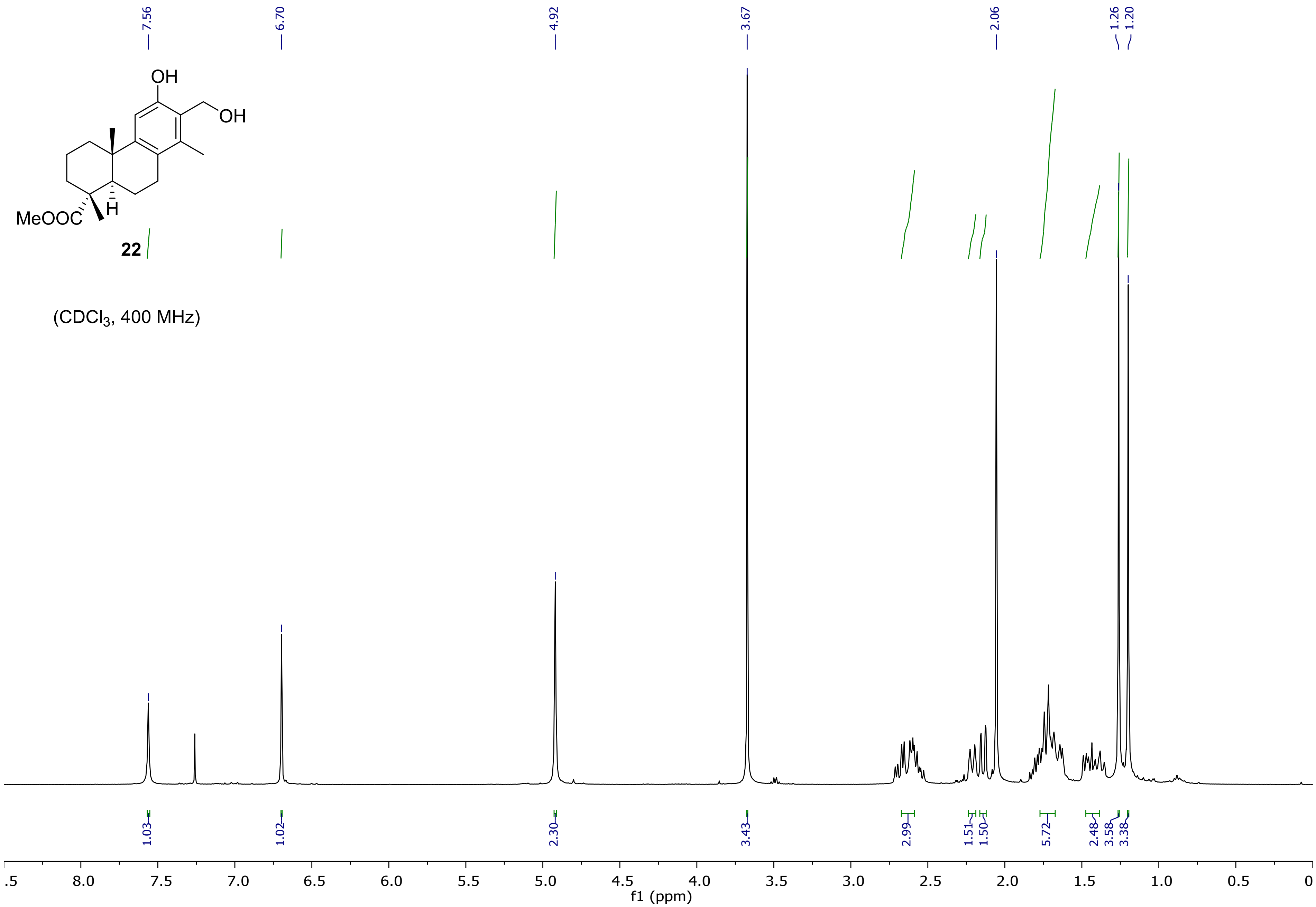
7.56
6.70
4.92
3.67
2.06
1.26
1.20
OH
OH
MeOOC
H
22
(CDCl$_3$, 400 MHz)
1.03
1.02
2.30
3.43
2.99
1.51
1.50
5.72
2.48
3.58
3.38
.5
8.0
7.5
7.0
6.5
6.0
5.5
5.0
4.5
4.0
3.5
3.0
2.5
2.0
1.5
1.0
0.5
0
f1 (ppm)

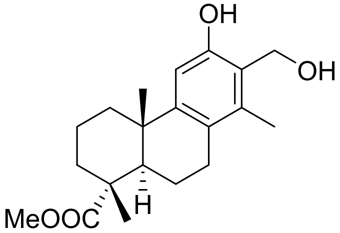

22

($CDCl_3$, 100 MHz)

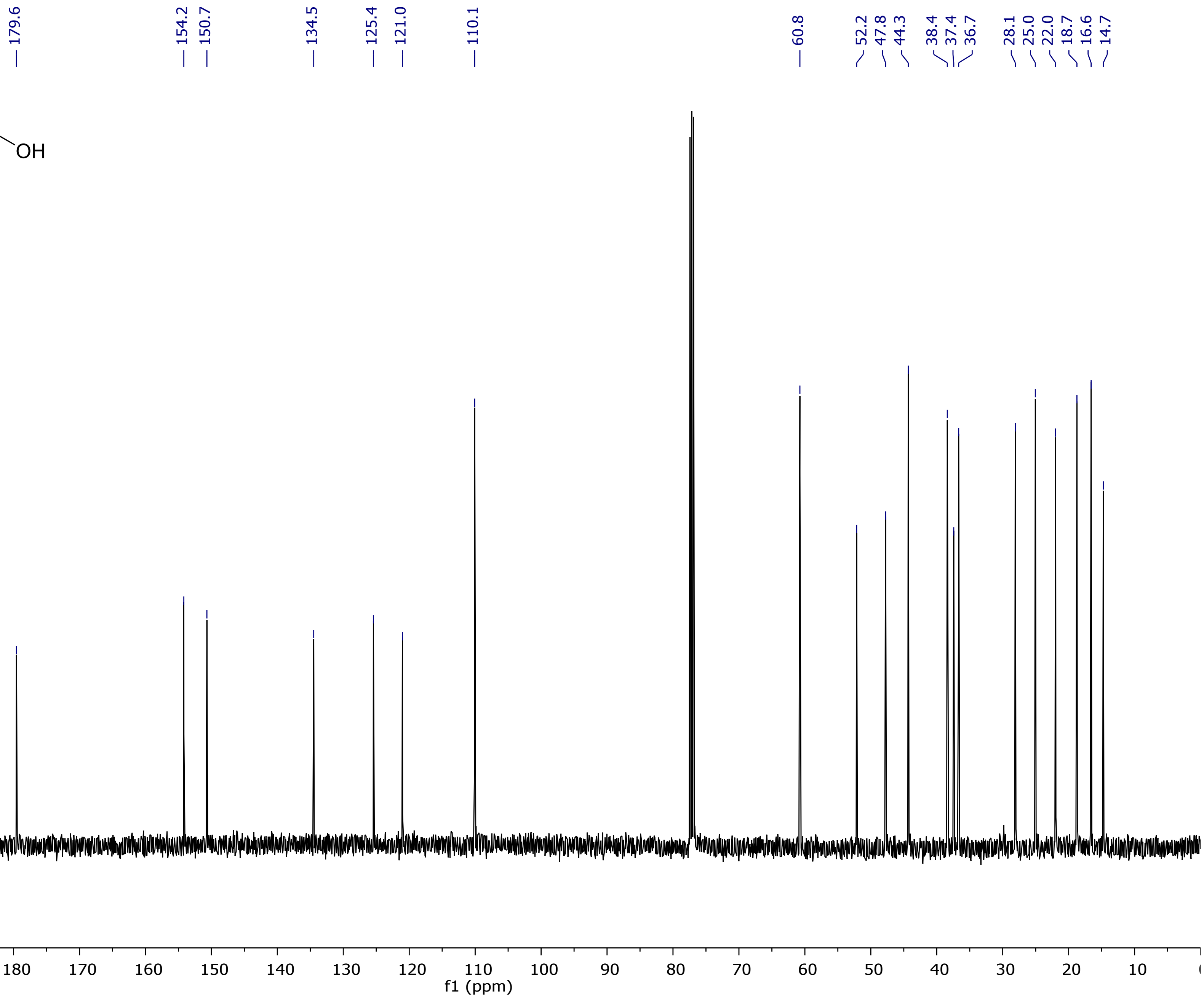

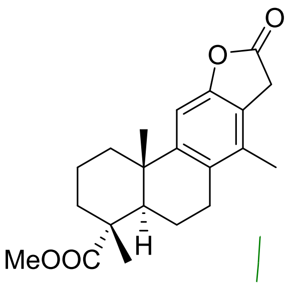

6

(CDCl$_3$, 400 MHz)

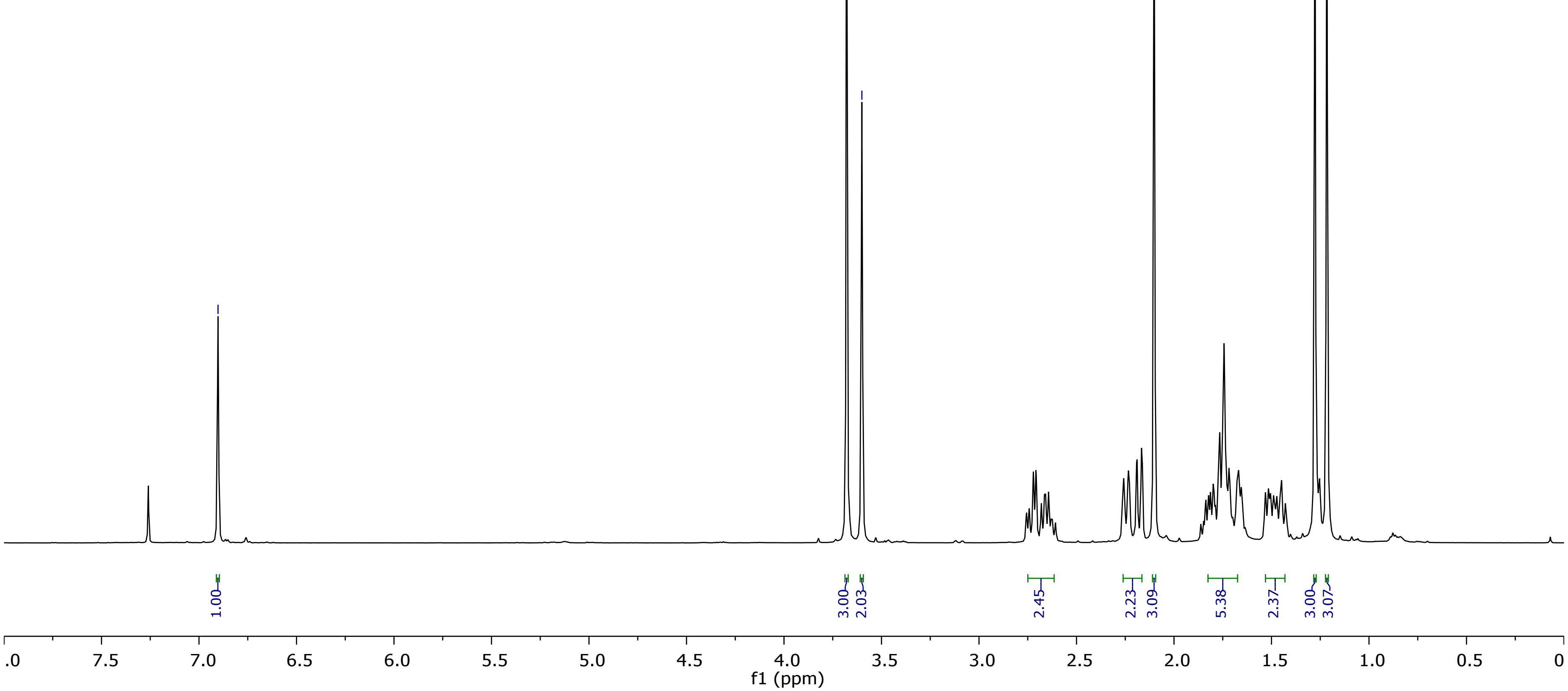

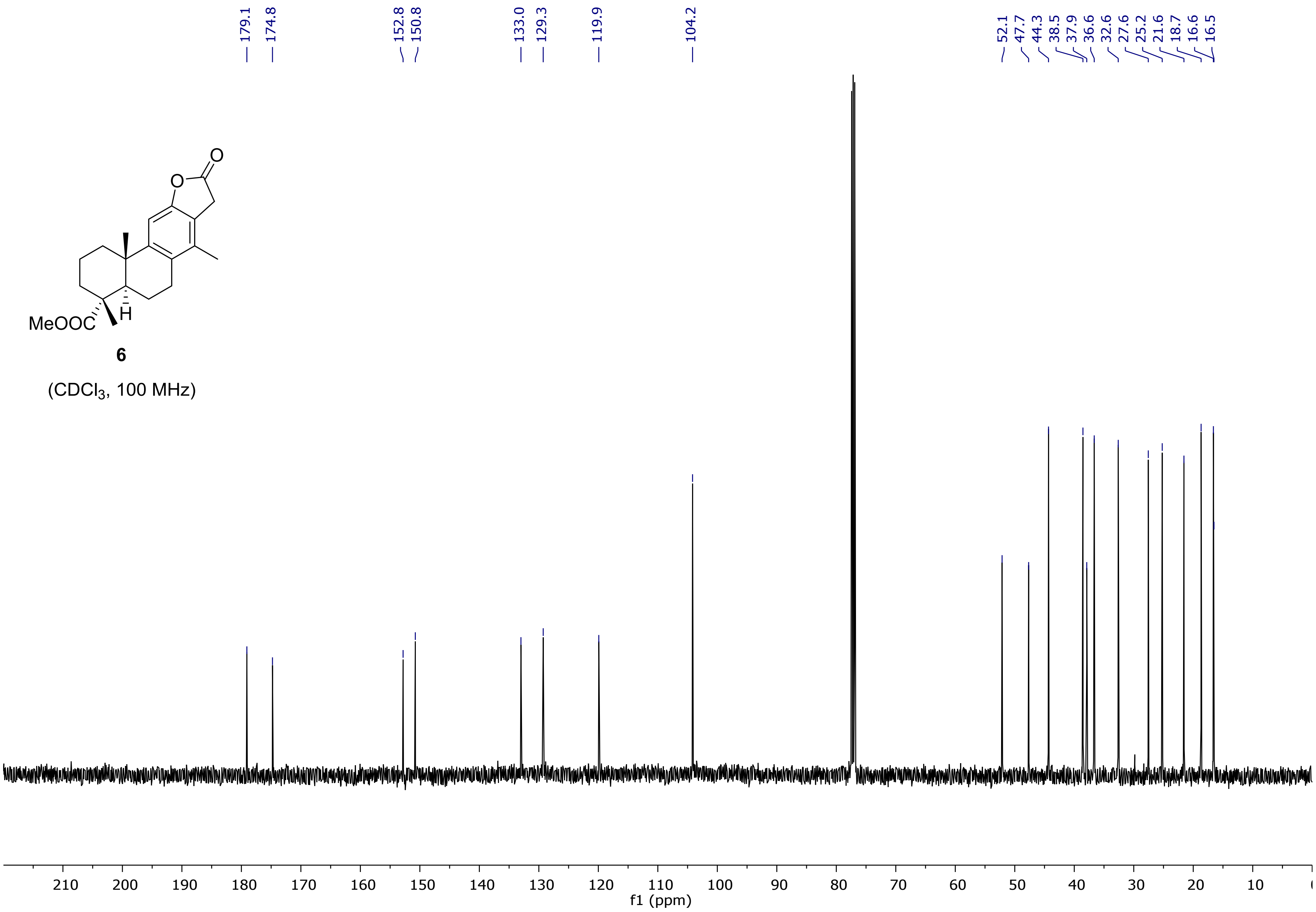

179.1
174.8
152.8
150.8
133.0
129.3
119.9
104.2
52.1
47.7
44.3
38.5
37.9
36.6
32.6
27.6
25.2
21.6
18.7
16.6
16.5
O
O
MeOOC
H
6
($CDCl_3$, 100 MHz)
210
200
190
180
170
160
150
140
130
120
110
100
90
80
70
60
50
40
30
20
10
f1 (ppm)
